# Approval of the National Rifle Association and political violence: findings from a nationally representative survey

**DOI:** 10.1186/s40621-026-00685-2

**Published:** 2026-05-14

**Authors:** Garen J. Wintemute, Yueju Li, Aaron B. Shev, Sonia L. Robinson, Elizabeth A. Tomsich, Mona A. Wright, Veronica A. Pear

**Affiliations:** 1 Department of Emergency Medicine, School of Medicine, UC Davis Medical Center, 2315 Stockton Blvd, CA 95817 Sacramento, USA; 2 Centers for Violence Prevention, University of California Davis, Sacramento, CA USA; 3 Department of Public Health Sciences, School of Medicine, UC Davis, Sacramento, CA USA

**Keywords:** Political violence, National Rifle Association, Domestic violent extremism, Firearm violence, Civil war, Firearm ownership, Racism, Hostile sexism, Homonegativity, Homophobia, Transphobia, Xenophobia, Antisemitism, Islamophobia, QAnon, Christian nationalism, Political parties, Political polarization, MAGA movement, Authoritarianism, Trait aggression, Conspiracism, Intimate partner violence

## Abstract

**Background:**

The National Rifle Association (NRA) is one of the most widely known social movement organizations in the United States (US). At a time of heightened concern for political violence, we assess individual-level associations between self-reported NRA approval and support for political violence, willingness to engage in political violence, and attitudes and beliefs linked to political violence.

**Methods:**

Findings are for respondents to Wave 2 (conducted May 18-June 8, 2023) of a nationally representative longitudinal survey; participants are members of Ipsos KnowledgePanel. Prevalences are reported as weighted percentages with 95% confidence intervals (CIs). Associations are expressed as adjusted prevalence differences (aPDs), measured in percentage points (pp), with p-values adjusted for the false discovery rate and reported as q-values.

**Results:**

The Wave 2 completion rate was 84.2%. There were 9,385 respondents, of whom 8,361 (89.1%) reported their level of NRA approval and are included here. Strong or very strong NRA approval was reported by 2,669 respondents (“NRA approvers”; 27.0%, 95% CI 25.7%, 28.2%), and 3,423 respondents (46.6%, 95% CI 45.1%, 48.0%) reported non-approval. Only half of NRA approvers (49.8%, 95% CI 47.2%, 52.5%) personally owned firearms. NRA approvers were more likely than non-approvers to support political violence and more willing to engage in it. For example, approvers were more than twice as likely as non-approvers to view violence as usually or always justified to advance at least 1 political objective (approvers 44.0%, 95% CI 41.4%, 46.6%; non-approvers 21.5%, 95% CI 19.6%, 23.4%; aPD 18.3pp, 95% CI 14.1pp, 22.5pp; q < 0.001) and to consider it very or extremely likely that they would shoot someone to advance a political objective (approvers 4.2%, 95% CI 2.9%, 5.5%; non-approvers 0.8%, 95% CI 0.4%, 1.2%; aPD 4.3pp, 95% CI 2.5pp, 6.1pp; q < 0.001). NRA approvers were more likely than non-approvers to endorse a wide array of beliefs and personal characteristics that have been associated with violence, including political violence.

**Conclusions:**

Approval of the NRA is associated with increased support for and willingness to engage in political violence, including lethal violence. These findings can help focus political violence prevention efforts in the United States.

**Supplementary Information:**

The online version contains supplementary material available at 10.1186/s40621-026-00685-2.

## Introduction

The National Rifle Association (NRA) is the principal organization of firearm owners in the United States (US) and is among the nation’s best-known social movement organizations [1–3]. Research suggests that the NRA fosters a particular social identity among its firearm owner members and supporters [4–10] that includes heightened willingness to take political action and to employ violence for purposes ranging from self-defense [7, 11] to insurrection [7, 12–14].

Studies comparing NRA members with nonmembers (or supporters with non-supporters), have found many important differences between them [6–8, 15]. For example, members own more firearms than nonmembers do [6]; among handgun owners, they are twice as likely to carry a loaded firearm most or all of the time when out in public [6]. Among firearm owners, NRA members are more than twice as likely as nonmembers to contact public officials on firearm policy topics [6].

The differences are not limited to firearm ownership and use. NRA members are more likely than nonmembers to be affiliated with the Republican Party, and among Republicans, they are more likely to describe themselves as very conservative [6]. They more frequently endorse statements of racial resentment [16], and racially resentful voters are more likely than others to support NRA-endorsed candidates for office [17]. NRA supporters are more likely than others to endorse expressions of xenophobia and ethno-nationalism [18]. In a recent study that identified 6 general classes of firearm owners, NRA supporters were over-represented in the class that ranked highest for firearm owner identity and had the highest levels of support for expressions of racism, sexism, homophobia, and lack of trust in government and the media [9].

Many of these characteristics have been associated at the individual level with increased support for and personal willingness to engage in political violence in our annual, nationally representative longitudinal survey of the US adult population [19–24]. At the population level, others have found an association between the prevalence of NRA membership and growth in right-wing extremist violence [18].

This constellation of findings raises the possibility that individual-level approval of the NRA is associated with support for and willingness to engage in political violence. We report here the findings of an investigation of that possibility, relying primarily on data collected in mid-2023’s Wave 2 of our survey. To provide context for those findings, we also report on associations between NRA approval and attitudes, beliefs, and behaviors that have been associated with political violence in our study cohort as a whole [19–24].

The motivation for this study is the concern that the US remains, in the view of experts in the field, at high risk for political violence in the near future [25–27]. Despite the NRA’s recent bankruptcy and scandal involving its leadership [28, 29], the organization likely remains capable of mobilizing large numbers of people to action in furtherance of shared political or social objectives. And more generally, the potential of a homogenous social network (the bonding form of social capital) to increase risk for committing political violence has been well documented [10, 30–32].

Given the research findings summarized here, it is important to know more about the characteristics of persons who approve of the NRA and might therefore participate in such a mobilization. If an association between NRA approval and support for political violence exists, such a mobilization might further increase risk for political violence in the US.

## Methods

Procedures for Wave 2 closely followed those for Wave 1 [19]. The questionnaire was designed by the authors and administered online in English and Spanish by the survey research firm Ipsos [33]. The study was reviewed by the University of California Davis Institutional Review Board (protocol 187125: exempt from full review, category 2, survey research). Written or verbal consent was not required; before participants accessed the questionnaire, they were provided informed consent language that concluded, “[by] continuing, you are agreeing to participate in this study.” The study is reported following American Association for Public Opinion Research guidelines [34].

### Participants

Survey participants were drawn from the Ipsos KnowledgePanel, an online research panel that has been widely used in population-based research, including studies of violence [35–38].

To establish a nationally representative panel, KnowledgePanel members are recruited on an ongoing basis through address-based probability sampling using data from the US Postal Service’s Delivery Sequence File [39, 40]. Recruitment into KnowledgePanel involves contact attempts by mail and telephone that are repeated if necessary. Recruited adults in households without internet access are provided a web-enabled device and free internet service. A modest incentive program seeks to encourage participation and promote participants’ retention in KnowledgePanel over time [39, 40].

A probability-proportional-to-size procedure was used to select a study-specific representative sample of KnowledgePanel members for Wave 1. All panel members who were aged 18 years and older in 2022 were eligible for selection. Invitations were sent by e-mail; automatic reminders were delivered to non-respondents by e-mail and telephone beginning 3 days later [39, 40].

The Wave 1 survey was fielded May 13 to June 2, 2022 [19]. It included a main sample, which had a completion rate of 53% (measured as completions/invitations), and oversamples of firearm owners, transgender people, combat veterans, and California residents to support preplanned supplemental analyses. Main sample respondents were older than nonrespondents and more frequently white, non-Hispanic; were more often married; had higher education and income; and were less likely to be working [19]. Altogether, Wave 1 included 12,947 respondents.

Invitations to participate in Wave 2 were sent to the 11,140 Wave 1 respondents (86.0%) who remained active members of KnowledgePanel on Wave 2’s launch date. (The remaining 1807 Wave 1 respondents had left the cohort through normal attrition.) Wave 2 was fielded from May 18 to June 8, 2023.

A final Wave 2 survey weight variable provided by Ipsos adjusted for the initial probability of selection into KnowledgePanel and for survey-specific nonresponse and over- or under-coverage using design weights with post-stratification raking ratio adjustments. As with the 2022 sample, the weighted 2023 sample is designed to be statistically representative of the noninstitutionalized adult population of the US as reflected in the March 2021 supplement of the Current Population Survey [39, 40].

### Measures

Sociodemographic data were collected by Ipsos from profiles created and maintained by KnowledgePanel members. Approval of the NRA was assessed using responses for “the National Rifle Association” to the Wave 1 (2022) question, “How much do you approve or disapprove of these named groups and organizations?” Response options were “do not approve,” “somewhat approve,” “strongly approve,” “very strongly approve,” “I don’t know enough about this group or organization to rate it,” and “I have never heard of this group or organization.”

Our primary outcome measures concerned political violence. Violence was represented in the questionnaire by “force or violence,” defined as “physical force strong enough that it could cause pain or injury to a person.” “Force or violence to advance an important political objective that you support” was used to denote political violence.

Respondents were asked about the extent to which they considered political violence to be justified “in general” and then about justification for its use to advance 19 specified political objectives (examples: “to return Donald Trump to the presidency this year,” “to stop police violence”).

Respondents who considered violence at least sometimes justified to advance at least 1 of these 19 objectives were asked about their personal willingness to engage in political violence: for 4 types of violence (to “damage property,” “threaten or intimidate a person,” “injure a person,” “kill a person”) and against 11 target populations (examples: “an elected federal or state government official,” “a police officer,” “a person who does not share your religion”) and by social context (examples: “on your own,” “as part of a group”).

All respondents were asked about the likelihood of their future use of firearms in a situation where they considered political violence justified (examples: “I will be armed with a gun”; “I will shoot someone with a gun”).

A wide array of respondent attitudes, beliefs, and behaviors was included in the analyses. These were grouped into 6 domains: democracy, authoritarianism, and elections; partisan identity and political ideology; hatred, fear, and enmity toward others; conspiracism and affiliation with extremist organizations and social movements; firearm ownership and use; and non-political aggression and violence.

Details of the definition and construction of all measures are in the Supplement (see Additional file 1), as are all related questionnaire items.

### Implementation

Ipsos translated the questionnaires into Spanish, and interpreting services staff at UC Davis Medical Center reviewed the translations. Members of KnowledgePanel (33 for Wave 1, 25 for Wave 2) participated in pretests of the English language version that were administered approximately 2 weeks before data collection began.

Respondents were randomized 1:1 to receive response options in order from negative to positive valence (e.g., from ‘do not agree’ to ‘very strongly agree’) or the reverse throughout the questionnaire. When an item presented multiple statements for respondents to consider, the presentation order was randomized unless ordering was necessary.

We employed unipolar response arrays without neutral midpoints (example: do not agree, somewhat agree, strongly agree, very strongly agree). There is disagreement on the use of neutral midpoints [41, 42]. We were persuaded by the studies reviewed by Chyung et al. [41], which suggest that midpoints facilitate selection of “a minimally acceptable response as soon as it is found,” known as satisficing. According to those authors, satisficing is particularly common when respondents are uncomfortable with the topics of the survey or under social desirability pressures, and both conditions apply here. Our analyses focus on responses above the “somewhat” or “sometimes” level to minimize the impact of satisficing on the results.

### Statistical analysis

Analyses were conducted using SAS version 9.4 (SAS Institute, Inc., Cary, NC). Respondents who strongly and very strongly approved of the NRA were combined for analysis. To generate prevalence estimates, we calculated weighted percentages and 95% confidence intervals (CI) using PROC SURVEYFREQ. To compute adjusted prevalence differences (aPDs) and 95% CIs, we defined outcomes dichotomously and used PROC SURVEYREG, employing robust standard errors to correct for design effects and heteroskedasticity in binary outcomes and weights to account for the complex survey design. We employed a model previously used for our study of the association between firearm ownership and political violence [21] that was based on concordance with theory, findings from prior research, and fit statistics and included respondent age, race and ethnicity, sex, income, education, census division, marital status, homeownership, political ideology, rurality, alcohol consumption, military service, and history of non-traffic arrest. aPDs are measured in absolute percentage points (pp).

We controlled for multiple comparisons by bounding the false discovery rate (FDR) using the Benjamini-Hochberg method [43]. The adjusted p-values are known as FDR-adjusted (or FDR-corrected) p-values or as q-values [44]; we employ the latter term here. For a given value of the false discovery rate, Q (Q = 0.05 was chosen here), the q-values are chosen so that, of comparisons that reject the null hypothesis (decided by q < α), the expected proportion of these comparisons that incorrectly reject the null hypothesis is at most Q.

The tables include findings for 3 categories of NRA approval: strongly/very strongly approve, somewhat approve, and do not approve. For simplicity, the text presents findings just for the first and third of these categories, described as “approvers” and “non-approvers,” respectively.

## Results

Of 11,140 panel members invited to participate in Wave 2, 9385 completed the survey, yielding an 84.2% completion rate. The median survey completion time was 25 min (interquartile range, 18.6 min). Item non-response ranged from 0.3% to 2.4%; 1 item had a non-response percentage above 2.0% (see Supplement, Additional file 1).

### Sample characteristics

After weighting, half of the respondents to the Wave 2 survey (51.1%, 95% CI 49.7%, 52.5%) were female; 62.7% (95% CI 61.2%, 64.1%) were white, non-Hispanic (see Supplement, Additional file 1, Table S1). The weighted mean (SD) respondent age was 57.0 (16.5) years. Compared with Wave 2 nonrespondents, respondents were older; more frequently male, non-Hispanic white, and married; and less frequently working full-time (see Supplement, Additional file 1, Table S2).

The analytic sample for this study comprised the 8,361 Wave 2 respondents (89.1% of the 9385 who completed the survey) who in Wave 1 reported their level of approval of the National Rifle Association: do not approve (*n* = 3,423, 46.6%, 95% CI 45.1%, 48.0%), somewhat approve (*n* = 2,269, 26.5%, 95% CI 25.2%, 27.7%), and strongly or very strongly approve (*n* = 2669, 27.0%, 95% CI 25.7%, 28.2%). Respondents who reported “I don’t know enough about this group or organization to rate it” (*n* = 726, 11.9%, 95% CI 10.9%, 12.9%) or “I have never heard of this group or organization” (*n* = 191, 4.6%, 95% CI 3.8%, 5.3%) and those who did not answer the question (*n* = 107, 1.7%, 95% CI 1.3%, 2.1%) were excluded. Compared with included respondents, these respondents were younger and more frequently female; less frequently white, non-Hispanic and married; and had lower levels of education and income (see Supplement, Additional file 1, Table S1). Among included respondents, those who approved strongly or very strongly of the NRA were older, more frequently non-Hispanic white, less likely to have a bachelor’s or advanced degree, and had lower incomes than did respondents who did not approve (see Supplement, Additional file 1, Table S1).

### Firearm ownership and use

Perhaps surprisingly, only half of NRA approvers (but a higher proportion than of non-approvers) reported personal ownership of firearms (approvers 49.8%, 95% CI 47.2%, 52.5%; non-approvers 15.5%, 95% CI 14.3%, 16.6%; aPD 24.6pp, 95% CI 21.3pp, 28.0pp; q < 0.001) (Table 1). Nonowners without firearms at home accounted for 39.1% (95% CI 36.4%, 41.9%) of NRA approvers, and nonowners with firearms at home accounted for 11.0% (95% CI 9.0%, 13.1%). Among firearm owners, NRA approvers were more likely to own assault-type rifles, to have purchased a firearm recently, and to always or nearly always carry a loaded firearm when out in public (Table 1).

**Table 1:** NRA approval and firearm ownership and use

Condition	How Much Do You Approve or Disapprove of…the National Rifle Association?					
	Do Not Approve		Somewhat Approve		Strongly/Very Strongly Approve	
	Unweighted *n*	Weighted % (95% CI)	Unweighted *n*	Weighted % (95% CI)	Unweighted *n*	Weighted % (95% CI)
Ownership						
Nonowner without firearms at home	2093	76.2 (74.5, 77.8)	874	55.0 (52.3, 57.7)	641	39.1 (36.4, 41.9)
Nonowner with firearms at home	211	8.4 (7.0, 9.7)	179	12.8 (10.8, 14.8)	146	11.0 (9.0, 13.1)
Owner	1117	15.5 (14.3, 16.6)	1201	32.2 (29.9, 34.5)	1835	49.8 (47.2, 52.5)
aPD (95% CI; q-value)	Referent		10.8 (8.2, 13.5; <0.001 )		24.6 (21.3, 28.0; <0.001 )	
**Asked of owners only**						
Type(s) of firearm owned						
Handgun only	320	32.2 (28.6, 35.7)	289	28.0 (24.5, 31.4)	340	19.6 (17.2, 21.9)
Other Rifle	493	39.5 (36.1, 43.0)	521	40.7 (37.0, 44.4)	814	43.2 (40.2, 46.3)
Other	153	13.1 (10.8, 15.4)	170	14.9 (12.3, 17.6)	194	9.8 (8.2, 11.3)
Assault-type rifle	134	15.2 (12.4, 18.0)	191	16.4 (13.9, 18.9)	446	27.4 (24.5, 30.4)
aPD (95% CI; q-value)	Referent		-2.0 (-6.1, 2.1; 0.45)		7.8 (2.9, 12.7; 0.003)	
Recency of firearm purchase						
Purchases only 2019 or earlier	891	75.1 (71.6, 78.6)	894	71.6 (68.3, 74.9)	1154	61.9 (59.0, 64.9)
Purchases 2020 or later	221	24.9 (21.4, 28.4)	297	28.4 (25.1, 31.7)	643	38.1 (35.1, 41.0)
aPD (95% CI; q-value)	Referent		0.2 (-4.9, 5.2; 0.95)		9.7 (4.2, 15.1; 0.001)	
Carrying in public						
Never or not often at all	1001	85.9 (83.0, 88.9)	984	81.0 (78.3, 83.7)	1257	66.8 (63.9, 69.7)
Less than half, about half, or more than half the time	74	9.4 (6.8, 12.1)	142	12.4 (10.2, 14.6)	344	20.0 (17.5, 22.4)
All or nearly all the time	42	4.6 (3.1, 6.2)	73	6.6 (4.9, 8.4)	224	13.3 (11.0, 15.5)
aPD (95% CI; q-value)	Referent		0.6 (-2.0, 3.3; 0.72)		6.6 (3.3, 9.9; <0.001 )	

Adjusted prevalence differences (aPDs) are absolute percentage point (pp) differences for the “owner,” “assault-type rifle,” and “all or nearly all the time” responses. Models are adjusted for age (continuous), race and ethnicity (7 categories: white, non-Hispanic; Black, non-Hispanic; Hispanic, any race; Asian American/Pacific Islander, non-Hispanic; American Indian/Alaska native, non-Hispanic; 2 + races, non-Hispanic; some other race, non-), gender (3 categories: male, female, other), income (7 categories: <$10,000, $10,000-$24,999, $25,000-$49,999, $50,000-$74,999, $75,000-$99,999, $100,000-$149,999, ≥$150,000), education (5 categories: no high school diploma or GED, high school graduate/GED, some college or associate’s degree, bachelor’s degree, master’s degree or higher), Census division (9 categories: New England, Middle Atlantic, South Atlantic, East North Central, East South Central, West North Central, West South Central, Mountain, Pacific), marital status (currently married, 2 categories: no, yes), homeownership (2 categories: no, yes), political ideology (conservative, 2 categories: no, yes), rurality (urban residence, 2 categories: no, yes), alcohol consumption (drinks/week, 3 categories: 0, 1–10, ≥ 11), military service (2 categories: no, yes), and history of non-traffic arrest (2 categories: no, yes)

For a given value of the false discovery rate, Q, the q-values are chosen so that, of comparisons that reject the null hypothesis (decided by q < α), the expected proportion of these comparisons that incorrectly reject the null hypothesis is at most Q. Item non-responses are not reported in the tables but are included in the prevalence calculations

### Political violence

NRA approvers were more likely than non-approvers to agree strongly or very strongly with 3 statements endorsing the use of violence to effect political and social change (Table 2), including “our American way of life is disappearing so fast that we may have to use force to save it” (approvers 25.2%, 95% CI 22.9%, 27.5%; non-approvers 5.6%, 95% CI 4.5%, 6.8%; aPD 17.7pp, 95% CI 14.3pp, 21.2pp; q < 0.001). They also more frequently agreed strongly or very strongly that “in the next few years, there will be civil war in the United States” (approvers 9.1%, 95% CI 7.5%, 10.7%; non-approvers 3.7%, 95% CI 2.8%, 4.7%; aPD 5.7pp, 95% CI 3.3pp, 8.2pp; q < 0.001) and that “the United States needs a civil war to set things right” (approvers 6.8%, 95% CI 5.4%, 8.2%; non-approvers 1.5%, 95% CI 1.0%, 2.1%; aPD 5.5pp, 95% CI 3.3pp, 7.7pp; q < 0.001).

**Table 2:** NRA approval and beliefs concerning violence to effect social change and a possible civil war

Statement	How Much Do You Approve or Disapprove of…the National Rifle Association?					
	Do Not Approve		Somewhat Approve		Strongly/Very Strongly Approve	
	Unweighted *n*	Weighted % (95% CI)	Unweighted *n*	Weighted % (95% CI)	Unweighted *n*	Weighted % (95% CI)
If elected leaders will not protect American democracy, the people must do it themselves, even if it requires taking violent actions.						
Do not agree	2712	76.6 (74.6, 78.6)	1454	61.5 (58.7, 64.3)	1123	42.3 (39.7, 44.9)
Somewhat agree	540	17.6 (15.8, 19.4)	626	29.4 (26.7, 32.1)	993	36.1 (33.6, 38.6)
Strongly or very strongly agree	150	5.1 (4.0, 6.1)	171	8.1 (6.4, 9.7)	514	19.7 (17.5, 21.8)
aPD (95% CI; q-value)	Referent		2.6 (0.2, 4.9; 0.04)		13.3 (10.4, 16.1; <0.001 )	
Our American way of life is disappearing so fast that we may have to use force to save it.						
Do not agree	2775	78.6 (76.7, 80.5)	1411	58.4 (55.5, 61.2)	1009	35.9 (33.4, 38.4)
Somewhat agree	474	15.1 (13.5, 16.8)	655	31.2 (28.5, 33.9)	997	37.2 (34.7, 39.8)
Strongly or very strongly agree	158	5.6 (4.5, 6.8)	189	9.6 (7.9, 11.4)	633	25.2 (22.9, 27.5)
aPD (95% CI; q-value)	Referent		2.9 (0.3, 5.5; 0.04)		17.7 (14.3, 21.2; <0.001 )	
Because things have gotten so far off track, true American patriots may have to resort to violence in order to save our country.						
Do not agree	3063	87.0 (85.4, 88.7)	1736	72.7 (70.0, 75.4)	1425	51.6 (49.0, 54.2)
Somewhat agree	247	8.5 (7.1, 9.9)	408	20.6 (18.1, 23.0)	823	31.2 (28.7, 33.6)
Strongly or very strongly agree	96	3.8 (2.8, 4.8)	111	5.8 (4.3, 7.3)	388	15.5 (13.6, 17.4)
aPD (95% CI; q-value)	Referent		1.3 (-0.8, 3.4; 0.22)		11.1 (8.3, 13.9; <0.001 )	
In the next few years, there will be civil war in the United States.						
Do not agree	2453	70.6 (68.5, 72.6)	1598	67.6 (64.8, 70.3)	1519	55.0 (52.3, 57.6)
Somewhat agree	838	25.7 (23.7, 27.7)	564	27.2 (24.7, 29.8)	891	35.9 (33.4, 38.5)
Strongly or very strongly agree	106	3.7 (2.8, 4.7)	84	5.2 (3.6, 6.8)	226	9.1 (7.5, 10.7)
aPD (95% CI; q-value)	Referent		1.5 (-0.5, 3.5; 0.14)		5.7 (3.3, 8.2; <0.001 )	
The United States needs a civil war to set things right.						
Do not agree	3224	93.2 (92.0, 94.5)	2013	86.4 (84.1, 88.7)	2034	75.8 (73.5, 78.1)
Somewhat agree	137	5.2 (4.1, 6.4)	183	9.6 (7.7, 11.5)	443	17.4 (15.3, 19.4)
Strongly or very strongly agree	40	1.5 (1.0, 2.1)	55	4.0 (2.5, 5.5)	162	6.8 (5.4, 8.2)
aPD (95% CI; q-value)	Referent		2.4 (0.5, 4.4; 0.02)		5.5 (3.3, 7.7; <0.001 )	

Adjusted prevalence differences (aPDs) are absolute percentage point (pp) differences for “strongly or very strongly agree” responses. Models are adjusted for age (continuous), race and ethnicity (7 categories: white, non-Hispanic; Black, non-Hispanic; Hispanic, any race; Asian American/Pacific Islander, non-Hispanic; American Indian/Alaska native, non-Hispanic; 2 + races, non-Hispanic; some other race, non-), gender (3 categories: male, female, other), income (7 categories: <$10,000, $10,000-$24,999, $25,000-$49,999, $50,000-$74,999, $75,000-$99,999, $100,000-$149,999, ≥$150,000), education (5 categories: no high school diploma or GED, high school graduate/GED, some college or associate’s degree, bachelor’s degree, master’s degree or higher), Census division (9 categories: New England, Middle Atlantic, South Atlantic, East North Central, East South Central, West North Central, West South Central, Mountain, Pacific), marital status (currently married, 2 categories: no, yes), homeownership (2 categories: no, yes), political ideology (conservative, 2 categories: no, yes), rurality (urban residence, 2 categories: no, yes), alcohol consumption (drinks/week, 3 categories: 0, 1–10, ≥ 11), military service (2 categories: no, yes), and history of non-traffic arrest (2 categories: no, yes)

For a given value of the false discovery rate, Q, the q-values are chosen so that, of comparisons that reject the null hypothesis (decided by q < α), the expected proportion of these comparisons that incorrectly reject the null hypothesis is at most Q. Item non-responses are not reported in the tables but are included in the prevalence calculations

NRA approvers were more likely than non-approvers to view violence as usually or always justified to advance at least 1 of 19 specific political objectives (approvers 44.0%, 95% CI 41.4%, 46.6%; non-approvers 21.5%, 95% CI 19.6%, 23.4%; aPD 18.3pp, 95% CI 14.1pp, 22.5pp; q < 0.001) and to advance 16 of those 19 objectives considered individually (Tables 3 and 4). Examples include violence “to return Donald Trump to the presidency this year” (i.e., in 2023; approvers 11.0%, 95% CI 9.3%, 12.8%; non-approvers 2.8%, 95% CI 2.0%, 3.6%; aPD 8.0pp, 95% CI 5.2pp, 10.8pp; q < 0.001) and “to stop illegal immigration” (approvers 25.7%, 95% CI 23.4%, 28.0%; non-approvers 4.2%, 95% CI 3.3%, 5.1%; aPD 17.5pp, 95% CI 14.3pp, 20.7pp; q < 0.001). The 3 exceptions were violence “to prevent discrimination based on race or ethnicity,” “to protect the environment or stop climate change,” and “to oppose Americans who do not share my beliefs.”

**Table 3:** NRA approval and justification for political violence “in general” and to advance specific political objectives

What do you think about the use of force or violence in the following situations?	How Much Do You Approve or Disapprove of…the National Rifle Association?					
	Do Not Approve		Somewhat Approve		Strongly/Very Strongly Approve	
	Unweighted *n*	Weighted % (95% CI)	Unweighted *n*	Weighted % (95% CI)	Unweighted *n*	Weighted % (95% CI)
In general…to advance an important political objective that you support						
Never justified	2857	79.7 (77.8, 81.6)	1869	78.4 (75.8, 80.9)	2097	77.6 (75.3, 79.9)
Sometimes justified	533	18.9 (17.1, 20.8)	370	19.3 (16.8, 21.8)	505	18.8 (16.7, 20.9)
Usually or always justified	26	1.0 (0.5, 1.5)	26	2.1 (1.2, 3.1)	57	3.2 (2.0, 4.4)
aPD (95% CI; q-value)	Referent		1.3 (0.1, 2.5; 0.06)		2.8 (1.1, 4.6; 0.004)	
Usually or always justified to advance at least 1 of 19 objectives	671	21.5 (19.6, 23.4)	596	27.5 (24.9, 30.1)	1194	44.0 (41.4, 46.6)
aPD (95% CI; q-value)	Referent		3.8 (0.2, 7.5; 0.07)		18.3 (14.1, 22.5; <0.001 )	
To return Donald Trump to the presidency this year [in 2023]						
Never justified	3292	95.2 (94.1, 96.3)	2101	89.6 (87.5, 91.8)	2213	80.6 (78.4, 82.9)
Sometimes justified	46	1.9 (1.1, 2.6)	85	5.1 (3.5, 6.7)	178	7.4 (5.9, 8.9)
Usually or always justified	73	2.8 (2.0, 3.6)	68	4.6 (3.1, 6.2)	253	11.0 (9.3, 12.8)
aPD (95% CI; q-value)	Referent		1.6 (-0.4, 3.7; 0.15)		8.0 (5.2, 10.8; <0.001 )	
To stop an election from being stolen						
Never justified	2910	84.3 (82.7, 86.0)	1776	75.8 (73.2, 78.4)	1766	67.3 (64.8, 69.8)
Sometimes justified	369	11.2 (9.8, 12.6)	359	17.0 (14.6, 19.3)	539	19.3 (17.2, 21.4)
Usually or always justified	126	4.0 (3.1, 4.9)	120	6.7 (5.2, 8.3)	343	12.7 (11.0, 14.4)
aPD (95% CI; q-value)	Referent		1.9 (-0.3, 4.2; 0.12)		7.5 (4.6, 10.3; <0.001 )	
To stop people who do not share my beliefs from voting						
Never justified	3309	95.4 (94.3, 96.5)	2166	91.8 (89.8, 93.9)	2494	90.5 (88.6, 92.4)
Sometimes justified	61	2.6 (1.7, 3.4)	65	5.7 (3.9, 7.5)	86	4.6 (3.2, 5.9)
Usually or always justified	38	1.8 (1.1, 2.5)	25	1.9 (1.0, 2.9)	69	4.2 (2.9, 5.6)
aPD (95% CI; q-value)	Referent		0.5 (-1.1, 2.1; 0.61)		2.9 (0.5, 5.3; 0.03)	
To prevent discrimination based on race or ethnicity						
Never justified	2502	69.8 (67.7, 71.9)	1717	72.0 (69.3, 74.7)	2003	72.3 (69.8, 74.8)
Sometimes justified	693	21.9 (20.1, 23.8)	437	21.0 (18.6, 23.5)	444	17.5 (15.5, 19.6)
Usually or always justified	213	8.0 (6.7, 9.4)	102	6.4 (4.8, 8.0)	202	9.4 (7.7, 11.1)
aPD (95% CI; q-value)	Referent		0.0 (-2.3, 2.4; 0.98)		3.7 (0.9, 6.4; 0.02)	
To preserve an American way of life based on Western European traditions						
Never justified	3036	89.8 (88.5, 91.1)	1759	77.1 (74.6, 79.6)	1696	65.3 (62.8, 67.8)
Sometimes justified	290	7.6 (6.4, 8.7)	405	16.5 (14.4, 18.6)	674	23.2 (21.1, 25.4)
Usually or always justified	81	2.4 (1.7, 3.1)	91	5.8 (4.2, 7.5)	264	10.4 (8.6, 12.1)
aPD (95% CI; q-value)	Referent		3.0 (0.9, 5.2; 0.01)		7.9 (5.3, 10.6; <0.001 )	
To preserve the American way of life l believe in						
Never justified	2759	81.9 (80.1, 83.6)	1497	66.8 (64.1, 69.5)	1279	51.4 (48.8, 54.0)
Sometimes justified	488	12.8 (11.3, 14.3)	608	24.7 (22.3, 27.1)	934	32.3 (29.8, 34.7)
Usually or always justified	159	5.0 (4.0, 6.0)	145	7.8 (6.1, 9.6)	432	15.5 (13.6, 17.4)
aPD (95% CI; q-value)	Referent		1.9 (-0.3, 4.1; 0.12)		9.5 (6.5, 12.4; <0.001 )	
To oppose Americans who do not share my beliefs						
Never justified	3209	91.7 (90.3, 93.1)	2101	88.9 (86.7, 91.1)	2389	87.0 (85.0, 89.1)
Sometimes justified	149	5.6 (4.5, 6.7)	119	8.2 (6.3, 10.2)	181	7.9 (6.4, 9.5)
Usually or always justified	45	2.2 (1.4, 3.0)	32	2.2 (1.2, 3.2)	76	4.2 (2.9, 5.5)
aPD (95% CI; q-value)	Referent		-0.1 (-1.9, 1.8; 0.98)		1.8 (-0.7, 4.3; 0.17)	
To oppose the government when it does not share my beliefs						
Never justified	3057	87.7 (86.2, 89.2)	1935	81.3 (78.8, 83.8)	2075	76.2 (73.8, 78.5)
Sometimes justified	288	9.9 (8.5, 11.3)	265	14.3 (12.1, 16.6)	439	16.6 (14.5, 18.6)
Usually or always justified	58	2.0 (1.4, 2.6)	54	3.7 (2.4, 5.0)	132	6.4 (5.0, 7.8)
aPD (95% CI; q-value)	Referent		1.7 (0.0, 3.3; 0.07)		4.7 (2.6, 6.8; <0.001 )	
To oppose the government when it tries to take private land for public purposes						
Never justified	2718	77.8 (75.9, 79.7)	1525	66.8 (64.1, 69.5)	1396	51.5 (48.9, 54.2)
Sometimes justified	562	17.2 (15.5, 18.8)	604	25.2 (22.8, 27.7)	884	32.7 (30.2, 35.2)
Usually or always justified	127	4.6 (3.6, 5.7)	123	7.3 (5.6, 8.9)	363	14.9 (13.0, 16.8)
aPD (95% CI; q-value)	Referent		2.5 (0.1, 4.9; 0.07)		10.6 (7.6, 13.7; <0.001 )	

Adjusted prevalence differences (aPDs) are absolute percentage point (pp) differences for “usually or always justified” responses. Models are adjusted for age (continuous), race and ethnicity (7 categories: white, non-Hispanic; Black, non-Hispanic; Hispanic, any race; Asian American/Pacific Islander, non-Hispanic; American Indian/Alaska native, non-Hispanic; 2 + races, non-Hispanic; some other race, non-), gender (3 categories: male, female, other), income (7 categories: <$10,000, $10,000-$24,999, $25,000-$49,999, $50,000-$74,999, $75,000-$99,999, $100,000-$149,999, ≥$150,000), education (5 categories: no high school diploma or GED, high school graduate/GED, some college or associate’s degree, bachelor’s degree, master’s degree or higher), Census division (9 categories: New England, Middle Atlantic, South Atlantic, East North Central, East South Central, West North Central, West South Central, Mountain, Pacific), marital status (currently married, 2 categories: no, yes), homeownership (2 categories: no, yes), political ideology (conservative, 2 categories: no, yes), rurality (urban residence, 2 categories: no, yes), alcohol consumption (drinks/week, 3 categories: 0, 1–10, ≥ 11), military service (2 categories: no, yes), and history of non-traffic arrest (2 categories: no, yes)

For a given value of the false discovery rate, Q, the q-values are chosen so that, of comparisons that reject the null hypothesis (decided by q < α), the expected proportion of these comparisons that incorrectly reject the null hypothesis is at most Q. Item non-responses are not reported in the tables but are included in the prevalence calculations

**Table 4:** NRA approval and justification for violence to advance 10 additional specific political objectives

What do you think about the use of force or violence in the following situations?	How Much Do You Approve or Disapprove of…the National Rifle Association?					
	Do Not Approve		Somewhat Approve		Strongly/Very Strongly Approve	
	Unweighted *n*	Weighted % (95% CI)	Unweighted *n*	Weighted % (95% CI)	Unweighted *n*	Weighted % (95% CI)
To stop voter fraud						
Never justified	2940	86.0 (84.4, 87.6)	1762	76.8 (74.3, 79.3)	1725	65.1 (62.5, 67.6)
Sometimes justified	300	8.4 (7.2, 9.7)	341	15.8 (13.6, 18.0)	534	19.7 (17.6, 21.8)
Usually or always justified	165	5.2 (4.2, 6.2)	148	6.7 (5.2, 8.2)	385	14.3 (12.5, 16.1)
aPD (95% CI; q-value)	Referent		1.4 (-0.9, 3.6; 0.3)		8.6 (5.6, 11.6; <0.001 )	
To stop voter intimidation						
Never justified	2440	72.1 (70.1, 74.1)	1604	70.4 (67.8, 73.0)	1691	65.2 (62.7, 67.7)
Sometimes justified	762	21.2 (19.4, 23.0)	503	21.8 (19.4, 24.1)	632	22.1 (19.9, 24.2)
Usually or always justified	204	6.3 (5.2, 7.5)	142	7.0 (5.4, 8.6)	323	11.9 (10.2, 13.7)
aPD (95% CI; q-value)	Referent		0.3 (-2.0, 2.6; 0.85)		5.5 (2.5, 8.5; <0.001 )	
To reinforce the police						
Never justified	2373	72.4 (70.5, 74.4)	1061	53.1 (50.3, 55.9)	813	34.7 (32.2, 37.3)
Sometimes justified	856	22.0 (20.2, 23.7)	952	35.6 (33.0, 38.2)	1213	42.7 (40.1, 45.3)
Usually or always justified	177	5.2 (4.2, 6.3)	238	10.6 (8.8, 12.5)	618	21.7 (19.6, 23.8)
aPD (95% CI; q-value)	Referent		5.0 (2.5, 7.4; <0.001 )		15.5 (12.3, 18.8; <0.001 )	
To stop police violence						
Never justified	2016	57.7 (55.5, 59.9)	1331	57.5 (54.7, 60.3)	1548	56.5 (53.9, 59.1)
Sometimes justified	1127	33.1 (31.0, 35.2)	773	34.1 (31.4, 36.8)	800	29.9 (27.4, 32.3)
Usually or always justified	265	9.0 (7.6, 10.3)	145	7.6 (5.9, 9.3)	296	12.8 (11.0, 14.6)
aPD (95% CI; q-value)	Referent		-0.5 (-3.0, 2.1; 0.8)		5.2 (2.2, 8.3; 0.002)	
To stop illegal immigration						
Never justified	2816	82.7 (81.0, 84.4)	1310	60.3 (57.5, 63.0)	977	39.8 (37.2, 42.4)
Sometimes justified	441	12.7 (11.2, 14.2)	697	28.2 (25.7, 30.6)	973	33.8 (31.3, 36.2)
Usually or always justified	147	4.2 (3.3, 5.1)	246	10.9 (9.0, 12.8)	698	25.7 (23.4, 28.0)
aPD (95% CI; q-value)	Referent		4.6 (2.2, 7.1; <0.001 )		17.5 (14.3, 20.7; <0.001 )	
To keep borders open						
Never justified	2842	81.3 (79.5, 83.1)	1811	78.1 (75.5, 80.6)	2079	76.1 (73.7, 78.4)
Sometimes justified	444	14.1 (12.5, 15.7)	334	15.8 (13.5, 18.0)	355	14.1 (12.2, 16.0)
Usually or always justified	119	4.2 (3.3, 5.2)	105	5.4 (4.0, 6.8)	211	9.0 (7.4, 10.7)
aPD (95% CI; q-value)	Referent		1.2 (-0.9, 3.4; 0.3)		5.3 (2.6, 8.0; <0.001 )	
To stop a protest						
Never justified	2839	82.8 (81.1, 84.5)	1525	68.2 (65.5, 70.8)	1535	57.4 (54.7, 60.0)
Sometimes justified	488	13.6 (12.1, 15.1)	652	26.8 (24.3, 29.2)	888	31.4 (29.0, 33.8)
Usually or always justified	80	3.3 (2.4, 4.2)	71	4.3 (3.0, 5.6)	220	10.3 (8.5, 12.2)
aPD (95% CI; q-value)	Referent		1.4 (-0.6, 3.5; 0.25)		7.4 (4.4, 10.3; <0.001 )	
To support a protest						
Never justified	2871	81.1 (79.2, 82.9)	1927	82.1 (79.7, 84.5)	2205	80.2 (78.0, 82.4)
Sometimes justified	438	15.0 (13.3, 16.7)	262	12.5 (10.5, 14.5)	330	13.5 (11.6, 15.3)
Usually or always justified	99	3.7 (2.8, 4.6)	62	4.7 (3.1, 6.3)	112	5.5 (4.2, 6.9)
aPD (95% CI; q-value)	Referent		1.4 (-0.6, 3.4; 0.25)		3.0 (0.7, 5.3; 0.02)	
To protect the environment or stop climate change						
Never justified	2628	74.1 (72.1, 76.1)	1856	77.2 (74.5, 79.8)	2266	81.3 (79.0, 83.6)
Sometimes justified	596	18.8 (17.0, 20.6)	307	16.5 (14.1, 18.9)	268	11.5 (9.8, 13.3)
Usually or always justified	185	6.8 (5.6, 8.0)	91	5.6 (4.1, 7.1)	116	6.4 (4.9, 8.0)
aPD (95% CI; q-value)	Referent		0.1 (-2.1, 2.2; 0.95)		1.5 (-1.1, 4.1; 0.3)	
To protect the rights of animals						
Never justified	2480	70.6 (68.5, 72.6)	1609	68.0 (65.3, 70.8)	1783	64.8 (62.2, 67.4)
Sometimes justified	703	21.8 (19.9, 23.7)	484	21.3 (18.9, 23.7)	569	21.9 (19.7, 24.2)
Usually or always justified	224	7.4 (6.2, 8.6)	165	10.2 (8.2, 12.2)	293	12.5 (10.7, 14.3)
aPD (95% CI; q-value)	Referent		3.7 (1.1, 6.4; 0.01)		6.8 (3.7, 9.9; <0.001 )	

Adjusted prevalence differences (aPDs) are absolute percentage point (pp) differences for “usually or always justified” responses. Models are adjusted for age (continuous), race and ethnicity (7 categories: white, non-Hispanic; Black, non-Hispanic; Hispanic, any race; Asian American/Pacific Islander, non-Hispanic; American Indian/Alaska native, non-Hispanic; 2 + races, non-Hispanic; some other race, non-), gender (3 categories: male, female, other), income (7 categories: <$10,000, $10,000-$24,999, $25,000-$49,999, $50,000-$74,999, $75,000-$99,999, $100,000-$149,999, ≥$150,000), education (5 categories: no high school diploma or GED, high school graduate/GED, some college or associate’s degree, bachelor’s degree, master’s degree or higher), Census division (9 categories: New England, Middle Atlantic, South Atlantic, East North Central, East South Central, West North Central, West South Central, Mountain, Pacific), marital status (currently married, 2 categories: no, yes), homeownership (2 categories: no, yes), political ideology (conservative, 2 categories: no, yes), rurality (urban residence, 2 categories: no, yes), alcohol consumption (drinks/week, 3 categories: 0, 1–10, ≥ 11), military service (2 categories: no, yes), and history of non-traffic arrest (2 categories: no, yes)

For a given value of the false discovery rate, Q, the q-values are chosen so that, of comparisons that reject the null hypothesis (decided by q < α), the expected proportion of these comparisons that incorrectly reject the null hypothesis is at most Q. Item non-responses are not reported in the tables but are included in the prevalence calculations

**Fig. 1:**
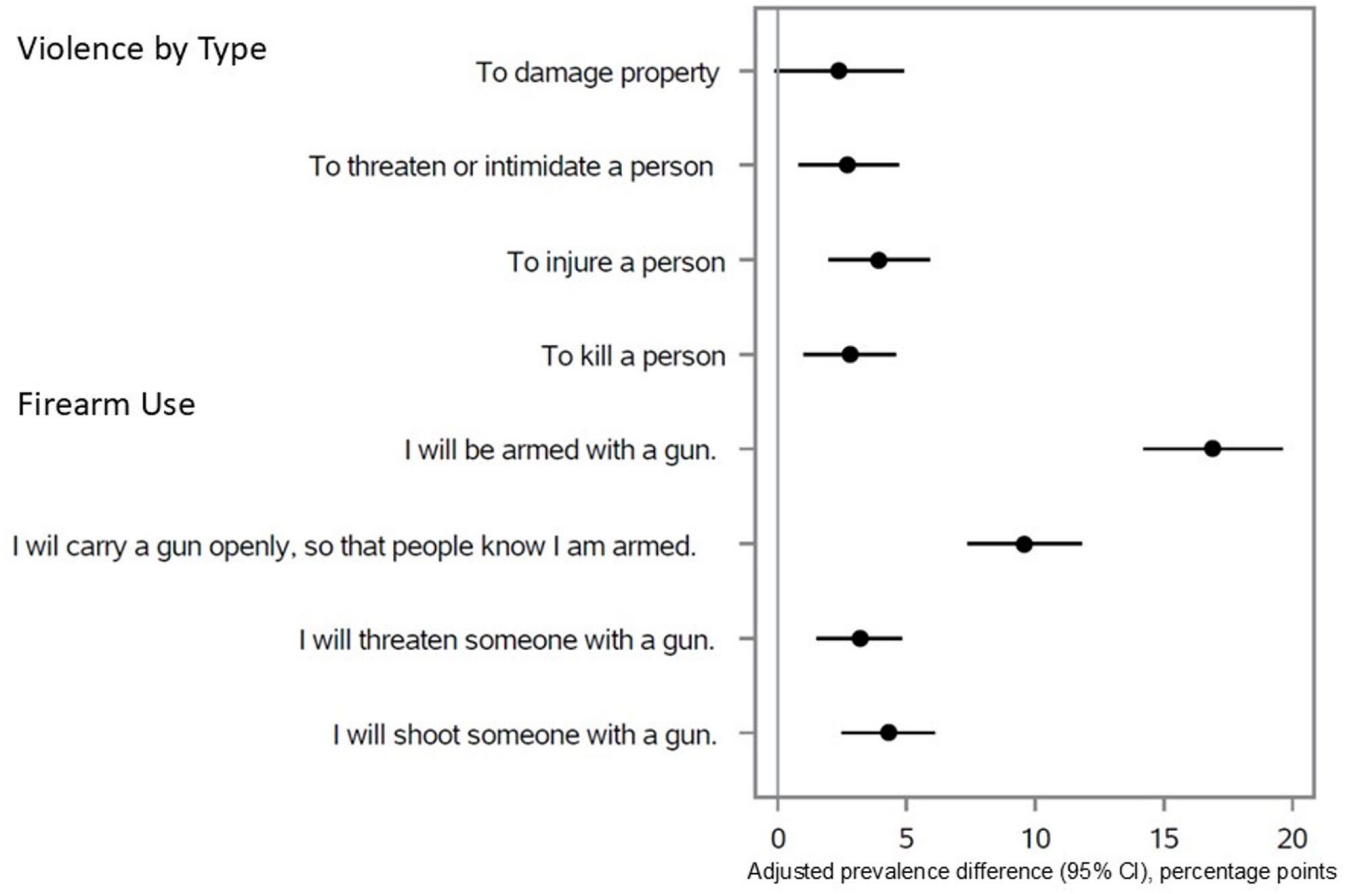
Differences between NRA approvers and non-approvers in willingness to engage in political violence and firearm use where political violence is viewed as justified. The text of the questionnaire items was as follows. Violence by type: In a situation where you think force or violence is justified to advance an important political objective, how willing would you personally be to use force or violence in each of these ways? Response options were not willing, somewhat willing, very willing, completely willing. Firearm use: Thinking now about the future and all the changes it might bring, how likely is it that you will use a gun in any of the following ways in the next few years—in a situation where you think force or violence is justified to advance an important political objective? Response options were not likely, somewhat likely, very likely, extremely likely. Adjusted prevalence differences are absolute percentage point (pp) differences for “very or completely willing” responses for violence by type and “very or extremely likely” responses for firearm use. Models are adjusted for age (continuous), race and ethnicity (7 categories: white, non-Hispanic; Black, non-Hispanic; Hispanic, any race; Asian American/Pacific Islander, non-Hispanic; American Indian/Alaska native, non-Hispanic; 2 + races, non-Hispanic; some other race, non-), gender (3 categories: male, female, other), income (7 categories: <$10,000, $10,000-$24,999, $25,000-$49,999, $50,000-$74,999, $75,000-$99,999, $100,000-$149,999, ≥$150,000), education (5 categories: no high school diploma or GED, high school graduate/GED, some college or associate’s degree, bachelor’s degree, master’s degree or higher), Census division (9 categories: New England, Middle Atlantic, South Atlantic, East North Central, East South Central, West North Central, West South Central, Mountain, Pacific), marital status (currently married, 2 categories: no, yes), homeownership (2 categories: no, yes), political ideology (conservative, 2 categories: no, yes), rurality (urban residence, 2 categories: no, yes), alcohol consumption (drinks/week, 3 categories: 0, 1–10, ≥ 11), military service (2 categories: no, yes), and history of non-traffic arrest (2 categories: no, yes)

Among those who considered political violence justified, NRA approvers were more likely than non-approvers to report that they were very or completely willing to engage personally in political violence against others (Table 5; Fig. 1), including “to injure a person” (approvers 4.2%, 95% CI 2.8%, 5.6%; non-approvers 0.9%, 95% CI 0.5%, 1.4%; aPD 3.9pp, 95% CI 2.0pp, 5.9pp; q < 0.001) and “to kill a person” (approvers 3.2%, 95% CI 2.0%, 4.4%; non-approvers 1.3%, 95% CI 0.6%, 2.0%; aPD 2.8pp, 95% CI 1.0pp, 4.6pp; q = 0.009). They were more willing to engage personally in violence against members of 3 of 9 specified target groups (Table 6): “an elected local government official” (approvers 3.1%, 95% CI 2.0%, 4.2%; non-approvers 0.9%, 95% CI 0.4%, 1.5%; aPD 3.4pp, 95% CI 1.5pp, 5.3pp; q = 0.007), “a public health official,” (approvers 2.9%, 95% CI 1.8%, 4.0%; non-approvers 0.9%, 95% CI 0.4%, 1.4%; aPD 2.7pp, 95% CI 0.8pp, 4.7pp; q = 0.03) and “a person who does not share your race or ethnicity” (approvers 2.9%, 95% CI 1.7%, 4.2%; non-approvers 1.0%, 95% CI 0.4%, 1.6%; aPD 2.9pp, 95% CI 1.0pp, 4.8pp; q = 0.03). They were more willing than non-approvers to engage in political violence on their own, as an individual (approvers 10.6%, 95% CI 8.9%, 12.3%; non-approvers 1.9%, 95% CI 1.2%, 2.5%; aPD 10.6pp, 95% CI 8.2pp, 12.9pp; q < 0.001), as part of a group, or as the organizer of a group (Table 7).

**Table 5:** NRA approval and personal willingness to engage in political violence, by type of violence

In a situation where you think force or violence is justified to advance an important political objective…How willing would *you personally* be to use force or violence in each of these ways?	How Much Do You Approve or Disapprove of…the National Rifle Association?					
	Do Not Approve		Somewhat Approve		Strongly/Very Strongly Approve	
	Unweighted *n*	Weighted % (95% CI)	Unweighted *n*	Weighted % (95% CI)	Unweighted *n*	Weighted % (95% CI)
To damage property						
Not asked the question	984	36.9 (34.5, 39.3)	371	22.3 (19.6, 24.9)	230	10.7 (9.0, 12.5)
Not willing	1473	49.5 (47.0, 51.9)	1338	65.1 (62.1, 68.2)	1828	74.2 (71.6, 76.7)
Somewhat willing	261	10.3 (8.8, 11.9)	159	9.4 (7.5, 11.4)	220	10.0 (8.2, 11.8)
Very or completely willing	56	2.8 (1.9, 3.8)	38	2.8 (1.5, 4.1)	89	4.9 (3.4, 6.4)
aPD (95% CI; q-value)	Referent		-0.1 (-1.9, 1.7; 0.93)		2.4 (-0.1, 4.9; 0.09)	
To threaten or intimidate a person						
Not asked the question	984	36.9 (34.5, 39.3)	371	22.3 (19.6, 24.9)	230	10.7 (9.0, 12.5)
Not willing	1545	53.2 (50.8, 55.7)	1321	64.7 (61.6, 67.7)	1802	73.1 (70.5, 75.6)
Somewhat willing	209	7.8 (6.3, 9.2)	177	9.6 (7.7, 11.4)	253	11.9 (9.8, 13.9)
Very or completely willing	32	1.6 (0.9, 2.2)	33	3.0 (1.5, 4.4)	80	4.0 (2.9, 5.1)
aPD (95% CI; q-value)	Referent		1.3 (-0.3, 3.0; 0.13)		2.7 (0.8, 4.7; 0.01)	
To injure a person						
Not asked the question	984	36.9 (34.5, 39.3)	371	22.3 (19.6, 24.9)	230	10.7 (9.0, 12.5)
Not willing	1628	55.8 (53.3, 58.2)	1373	66.8 (63.8, 69.8)	1883	76.0 (73.5, 78.6)
Somewhat willing	136	5.8 (4.5, 7.1)	128	8.0 (6.1, 9.8)	174	8.5 (6.7, 10.2)
Very or completely willing	23	0.9 (0.5, 1.4)	31	2.4 (1.2, 3.6)	71	4.2 (2.8, 5.6)
aPD (95% CI; q-value)	Referent		2.0 (0.4, 3.6; 0.03)		3.9 (2.0, 5.9; <0.001)	
To kill a person						
Not asked the question	984	36.9 (34.5, 39.3)	371	22.3 (19.6, 24.9)	230	10.7 (9.0, 12.5)
Not willing	1693	58.6 (56.1, 61.0)	1430	69.9 (67.0, 72.9)	1966	80.0 (77.6, 82.4)
Somewhat willing	65	2.6 (1.7, 3.4)	73	5.5 (3.8, 7.2)	108	5.7 (4.1, 7.3)
Very or completely willing	28	1.3 (0.6, 2.0)	28	1.7 (0.8, 2.5)	60	3.2 (2.0, 4.4)
aPD (95% CI; q-value)	Referent		1.1 (-0.1, 2.4; 0.1)		2.8 (1.0, 4.6; 0.009)	

Adjusted prevalence differences (aPDs) are absolute percentage point (pp) differences for “very or completely willing” responses. Models are adjusted for age (continuous), race and ethnicity (7 categories: white, non-Hispanic; Black, non-Hispanic; Hispanic, any race; Asian American/Pacific Islander, non-Hispanic; American Indian/Alaska native, non-Hispanic; 2 + races, non-Hispanic; some other race, non-), gender (3 categories: male, female, other), income (7 categories: <$10,000, $10,000-$24,999, $25,000-$49,999, $50,000-$74,999, $75,000-$99,999, $100,000-$149,999, ≥$150,000), education (5 categories: no high school diploma or GED, high school graduate/GED, some college or associate’s degree, bachelor’s degree, master’s degree or higher), Census division (9 categories: New England, Middle Atlantic, South Atlantic, East North Central, East South Central, West North Central, West South Central, Mountain, Pacific), marital status (currently married, 2 categories: no, yes), homeownership (2 categories: no, yes), political ideology (conservative, 2 categories: no, yes), rurality (urban residence, 2 categories: no, yes), alcohol consumption (drinks/week, 3 categories: 0, 1–10, ≥ 11), military service (2 categories: no, yes), and history of non-traffic arrest (2 categories: no, yes)

For a given value of the false discovery rate, Q, the q-values are chosen so that, of comparisons that reject the null hypothesis (decided by q < α), the expected proportion of these comparisons that incorrectly reject the null hypothesis is at most Q. Item non-responses are not reported in the tables but are included in the prevalence calculations

**Table 6:** NRA approval and personal willingness to engage in political violence, by target of violence

In a situation where you think force or violence is justified to advance an important political objective… How willing would you personally be to use force or violence against a person because they are…	How Much Do You Approve or Disapprove of…the National Rifle Association?					
	Do Not Approve		Somewhat Approve		Strongly/Very Strongly Approve	
	Unweighted *n*	Weighted % (95% CI)	Unweighted *n*	Weighted % (95% CI)	Unweighted *n*	Weighted % (95% CI)
An elected federal or state government official						
Not asked the question	984	36.9 (34.5, 39.3)	371	22.3 (19.6, 24.9)	230	10.7 (9.0, 12.5)
Not willing	1664	56.5 (54.1, 59.0)	1424	70.2 (67.2, 73.1)	1940	78.9 (76.5, 81.4)
Somewhat willing	93	4.7 (3.4, 6.0)	81	4.9 (3.4, 6.5)	131	6.1 (4.6, 7.7)
Very or completely willing	25	1.1 (0.5, 1.6)	20	1.7 (0.7, 2.7)	50	3.0 (1.8, 4.2)
aPD (95% CI; q-value)	Referent		1.0 (-0.6, 2.5; 0.31)		2.1 (0.2, 3.9; 0.08)	
An elected local government official						
Not asked the question	984	36.9 (34.5, 39.3)	371	22.3 (19.6, 24.9)	230	10.7 (9.0, 12.5)
Not willing	1684	57.5 (55.0, 60.0)	1441	70.8 (67.9, 73.7)	1941	79.1 (76.7, 81.5)
Somewhat willing	85	4.1 (2.9, 5.2)	70	4.4 (2.9, 5.8)	125	5.8 (4.4, 7.2)
Very or completely willing	18	0.9 (0.4, 1.5)	16	1.6 (0.5, 2.7)	52	3.1 (2.0, 4.2)
aPD (95% CI; q-value)	Referent		1.7 (0.0, 3.4; 0.13)		3.4 (1.5, 5.3; 0.007)	
An election worker, such as a poll worker or vote counter						
Not asked the question	984	36.9 (34.5, 39.3)	371	22.3 (19.6, 24.9)	230	10.7 (9.0, 12.5)
Not willing	1722	58.9 (56.5, 61.4)	1482	72.9 (70.1, 75.8)	2019	81.4 (79.1, 83.8)
Somewhat willing	42	2.2 (1.4, 3.0)	34	2.5 (1.4, 3.6)	61	3.7 (2.3, 5.0)
Very or completely willing	19	1.2 (0.5, 1.9)	15	1.5 (0.4, 2.7)	39	2.8 (1.6, 4.1)
aPD (95% CI; q-value)	Referent		0.4 (-1.1, 1.9; 0.7)		2.0 (-0.2, 4.1; 0.13)	
A public health official						
Not asked the question	984	36.9 (34.5, 39.3)	371	22.3 (19.6, 24.9)	230	10.7 (9.0, 12.5)
Not willing	1711	59.0 (56.5, 61.4)	1461	71.7 (68.8, 74.6)	1972	79.5 (77.0, 82.0)
Somewhat willing	50	2.4 (1.5, 3.3)	46	3.0 (1.8, 4.2)	96	5.6 (3.9, 7.3)
Very or completely willing	19	0.9 (0.4, 1.4)	21	2.3 (1.1, 3.6)	52	2.9 (1.8, 4.0)
aPD (95% CI; q-value)	Referent		2.0 (0.3, 3.7; 0.08)		2.7 (0.8, 4.7; 0.03)	
A member of the military or National Guard						
Not asked the question	984	36.9 (34.5, 39.3)	371	22.3 (19.6, 24.9)	230	10.7 (9.0, 12.5)
Not willing	1686	57.2 (54.7, 59.7)	1450	71.4 (68.6, 74.3)	1991	80.5 (78.1, 82.9)
Somewhat willing	74	4.0 (2.8, 5.2)	63	4.0 (2.8, 5.3)	86	5.1 (3.6, 6.7)
Very or completely willing	23	1.2 (0.6, 1.9)	15	1.4 (0.4, 2.4)	44	2.5 (1.4, 3.5)
aPD (95% CI; q-value)	Referent		0.8 (-0.6, 2.2; 0.36)		1.9 (0.2, 3.6; 0.08)	
A police officer						
Not asked the question	984	36.9 (34.5, 39.3)	371	22.3 (19.6, 24.9)	230	10.7 (9.0, 12.5)
Not willing	1630	54.9 (52.4, 57.4)	1429	69.6 (66.7, 72.6)	1982	79.4 (76.9, 81.9)
Somewhat willing	111	5.4 (4.1, 6.7)	78	5.4 (3.8, 7.1)	91	6.2 (4.4, 7.9)
Very or completely willing	43	2.1 (1.3, 2.9)	22	2.0 (0.8, 3.1)	48	2.6 (1.6, 3.6)
aPD (95% CI; q-value)	Referent		0.5 (-1.2, 2.1; 0.7)		1.9 (-0.1, 3.8; 0.13)	
A person who does not share your race or ethnicity						
Not asked the question	984	36.9 (34.5, 39.3)	371	22.3 (19.6, 24.9)	230	10.7 (9.0, 12.5)
Not willing	1722	59.3 (56.9, 61.8)	1459	71.0 (68.1, 74.0)	2010	80.9 (78.5, 83.3)
Somewhat willing	44	2.1 (1.2, 3.0)	51	4.0 (2.5, 5.5)	70	4.1 (2.7, 5.5)
Very or completely willing	16	1.0 (0.4, 1.6)	14	1.5 (0.4, 2.5)	40	2.9 (1.7, 4.2)
aPD (95% CI; q-value)	Referent		0.9 (-0.5, 2.3; 0.31)		2.9 (1.0, 4.8; 0.03)	
A person who does not share your religion						
Not asked the question	984	36.9 (34.5, 39.3)	371	22.3 (19.6, 24.9)	230	10.7 (9.0, 12.5)
Not willing	1719	58.7 (56.3, 61.2)	1480	72.8 (69.9, 75.7)	2014	81.0 (78.6, 83.4)
Somewhat willing	39	2.1 (1.2, 2.9)	37	3.2 (1.7, 4.7)	74	4.3 (2.9, 5.7)
Very or completely willing	22	1.5 (0.7, 2.2)	13	1.1 (0.3, 1.9)	38	2.8 (1.6, 4.1)
aPD (95% CI; q-value)	Referent		0.0 (-1.6, 1.6; 0.98)		1.6 (-0.8, 3.9; 0.31)	
A person who does not share your political beliefs						
Not asked the question	984	36.9 (34.5, 39.3)	371	22.3 (19.6, 24.9)	230	10.7 (9.0, 12.5)
Not willing	1682	57.3 (54.8, 59.7)	1454	71.4 (68.5, 74.3)	1990	80.6 (78.2, 82.9)
Somewhat willing	80	4.1 (2.9, 5.3)	64	4.3 (2.8, 5.8)	85	4.3 (3.1, 5.6)
Very or completely willing	19	1.1 (0.5, 1.6)	11	1.1 (0.2, 2.1)	41	3.0 (1.7, 4.3)
aPD (95% CI; q-value)	Referent		0.3 (-1.3, 1.9; 0.76)		2.0 (-0.2, 4.1; 0.13)	

Adjusted prevalence differences (aPDs) are absolute percentage point (pp) differences for “very or completely willing” responses. Models are adjusted for age (continuous), race and ethnicity (7 categories: white, non-Hispanic; Black, non-Hispanic; Hispanic, any race; Asian American/Pacific Islander, non-Hispanic; American Indian/Alaska native, non-Hispanic; 2 + races, non-Hispanic; some other race, non-), gender (3 categories: male, female, other), income (7 categories: <$10,000, $10,000-$24,999, $25,000-$49,999, $50,000-$74,999, $75,000-$99,999, $100,000-$149,999, ≥$150,000), education (5 categories: no high school diploma or GED, high school graduate/GED, some college or associate’s degree, bachelor’s degree, master’s degree or higher), Census division (9 categories: New England, Middle Atlantic, South Atlantic, East North Central, East South Central, West North Central, West South Central, Mountain, Pacific), marital status (currently married, 2 categories: no, yes), homeownership (2 categories: no, yes), political ideology (conservative, 2 categories: no, yes), rurality (urban residence, 2 categories: no, yes), alcohol consumption (drinks/week, 3 categories: 0, 1–10, ≥ 11), military service (2 categories: no, yes), and history of non-traffic arrest (2 categories: no, yes)

For a given value of the false discovery rate, Q, the q-values are chosen so that, of comparisons that reject the null hypothesis (decided by q < α), the expected proportion of these comparisons that incorrectly reject the null hypothesis is at most Q. Item non-responses are not reported in the tables but are included in the prevalence calculations

**Table 7:** NRA approval and personal willingness to engage in political violence, by social context of violence

You agreed that the use of force or violence could be justified to advance (one/some) of the political objectives we just discussed. In (that/those) (situation/situations), how willing would you personally be to…	How Much Do You Approve or Disapprove of…the National Rifle Association?					
	Do Not Approve		Somewhat Approve		Strongly/Very Strongly Approve	
	Unweighted *n*	Weighted % (95% CI)	Unweighted *n*	Weighted % (95% CI)	Unweighted *n*	Weighted % (95% CI)
Use force or violence as part of a group of people who share your beliefs						
Not asked the question	984	29.9 (27.8, 31.9)	371	18.2 (16.0, 20.4)	230	9.3 (7.8, 10.8)
Not willing	2111	59.6 (57.4, 61.8)	1598	67.4 (64.7, 70.1)	1811	67.4 (64.9, 69.9)
Sometimes willing	276	8.5 (7.1, 9.8)	252	11.8 (9.9, 13.7)	484	17.1 (15.2, 19.1)
Very or completely willing	43	1.6 (1.0, 2.2)	39	2.1 (1.2, 3.1)	119	5.0 (3.7, 6.3)
aPD (95% CI; q-value)	Referent		0.8 (-0.6, 2.2; 0.26)		4.1 (2.2, 6.1; <0.001 )	
Use force or violence on your own, as an individual						
Not asked the question	984	29.9 (27.8, 31.9)	371	18.2 (16.0, 20.4)	230	9.3 (7.8, 10.8)
Not willing	1940	55.9 (53.7, 58.2)	1400	60.2 (57.4, 63.0)	1566	58.5 (55.9, 61.1)
Sometimes willing	426	11.9 (10.4, 13.4)	395	15.9 (13.9, 17.9)	581	20.7 (18.6, 22.9)
Very or completely willing	64	1.9 (1.2, 2.5)	97	5.3 (3.8, 6.8)	269	10.6 (8.9, 12.3)
aPD (95% CI; q-value)	Referent		4.1 (2.2, 6.0; <0.001 )		10.6 (8.2, 12.9; <0.001 )	
Organize a group of people who share your beliefs to use force or violence						
Not asked the question	984	29.9 (27.8, 31.9)	371	18.2 (16.0, 20.4)	230	9.3 (7.8, 10.8)
Not willing	2239	63.2 (61.0, 65.3)	1706	71.3 (68.7, 74.0)	2027	74.1 (71.7, 76.5)
Sometimes willing	162	5.3 (4.2, 6.4)	151	7.5 (6.0, 9.1)	295	11.3 (9.5, 13.1)
Very or completely willing	29	1.2 (0.6, 1.7)	36	2.6 (1.4, 3.7)	92	4.2 (3.1, 5.3)
aPD (95% CI; q-value)	Referent		1.9 (0.6, 3.1; 0.005)		4.9 (3.1, 6.7; <0.001 )	

Adjusted prevalence differences (aPDs) are absolute percentage point (pp) differences for “very or completely willing” responses. Models are adjusted for age (continuous), race and ethnicity (7 categories: white, non-Hispanic; Black, non-Hispanic; Hispanic, any race; Asian American/Pacific Islander, non-Hispanic; American Indian/Alaska native, non-Hispanic; 2 + races, non-Hispanic; some other race, non-), gender (3 categories: male, female, other), income (7 categories: <$10,000, $10,000-$24,999, $25,000-$49,999, $50,000-$74,999, $75,000-$99,999, $100,000-$149,999, ≥$150,000), education (5 categories: no high school diploma or GED, high school graduate/GED, some college or associate’s degree, bachelor’s degree, master’s degree or higher), Census division (9 categories: New England, Middle Atlantic, South Atlantic, East North Central, East South Central, West North Central, West South Central, Mountain, Pacific), marital status (currently married, 2 categories: no, yes), homeownership (2 categories: no, yes), political ideology (conservative, 2 categories: no, yes), rurality (urban residence, 2 categories: no, yes), alcohol consumption (drinks/week, 3 categories: 0, 1–10, ≥ 11), military service (2 categories: no, yes), and history of non-traffic arrest (2 categories: no, yes)

For a given value of the false discovery rate, Q, the q-values are chosen so that, of comparisons that reject the null hypothesis (decided by q < α), the expected proportion of these comparisons that incorrectly reject the null hypothesis is at most Q. Item non-responses are not reported in the tables but are included in the prevalence calculations

### Firearms and political violence

NRA approvers were more likely than non-approvers (Table 8; Fig. 1) to consider it very or extremely likely that, in a future situation where they considered political violence justified, they would be “armed with a gun” (approvers 21.9%, 95% CI 19.9%, 23.9%; non-approvers 3.0%, 95% CI 2.3%, 3.7%; aPD 16.9pp, 95% CI 14.2pp, 19.6pp; q < 0.001), would “threaten someone with a gun” (approvers 3.6%, 95% CI 2.3%, 5.0%; non-approvers 0.8%, 95% CI 0.3%, 1.3%; aPD 3.2pp, 95% CI 1.5pp, 4.8pp; q < 0.001) and would “shoot someone with a gun (approvers 4.2%, 95% CI 2.9%, 5.5%; non-approvers 0.8%, 95% CI 0.4%, 1.2%; aPD 4.3pp, 95% CI 2.5pp, 6.1pp; q < 0.001).

**Table 8:** NRA approval and future firearm possession and use when political violence is perceived as justified

Thinking now about the future and all the changes it might bring, how likely is it that you will use a gun in any of the following ways in the next few years—in a situation where you think force or violence is justified to advance an important political objective?	How Much Do You Approve or Disapprove of…the National Rifle Association?					
	Do Not Approve		Somewhat Approve		Strongly/Very Strongly Approve	
	Unweighted *n*	Weighted % (95% CI)	Unweighted *n*	Weighted % (95% CI)	Unweighted *n*	Weighted % (95% CI)
I will be armed with a gun.						
Not likely	3029	90.0 (88.6, 91.4)	1637	74.6 (72.1, 77.1)	1384	55.6 (53.0, 58.3)
Somewhat likely	237	7.0 (5.7, 8.2)	374	16.1 (14.0, 18.2)	541	22.4 (20.0, 24.8)
Very or extremely likely	134	3.0 (2.3, 3.7)	238	9.3 (7.6, 11)	694	21.9 (19.9, 23.9)
aPD (95% CI; q-value)	Referent		4.4 (2.3, 6.5; <0.001 )		16.9 (14.2, 19.6; <0.001 )	
I will carry a gun openly, so that people know I am armed.						
Not likely	3258	95.6 (94.5, 96.6)	1972	87.5 (85.5, 89.6)	1906	73.5 (71.1, 75.9)
Somewhat likely	93	3.1 (2.2, 4.0)	213	9.6 (7.7, 11.4)	409	14.8 (12.9, 16.7)
Very or extremely likely	43	1.3 (0.8, 1.9)	62	2.9 (1.9, 3.9)	306	11.7 (9.9, 13.5)
aPD (95% CI; q-value)	Referent		1.4 (0.0, 2.9; 0.07)		9.6 (7.4, 11.8; <0.001 )	
I will threaten someone with a gun.						
Not likely	3356	97.7 (96.8, 98.5)	2202	96.7 (95.4, 98.0)	2485	93.1 (91.5, 94.8)
Somewhat likely	28	1.5 (0.8, 2.3)	31	2.3 (1.2, 3.5)	80	3.2 (2.2, 4.3)
Very or extremely likely	16	0.8 (0.3, 1.3)	15	0.9 (0.2, 1.6)	55	3.6 (2.3, 5.0)
aPD (95% CI; q-value)	Referent		0.5 (-0.5, 1.5; 0.34)		3.2 (1.5, 4.8; <0.001 )	
I will shoot someone with a gun.						
Not likely	3336	97.0 (96.1, 98.0)	2152	95.2 (93.8, 96.6)	2362	89.6 (87.8, 91.5)
Somewhat likely	45	2.2 (1.3, 3.1)	77	3.7 (2.5, 4.9)	173	6.1 (4.8, 7.5)
Very or extremely likely	21	0.8 (0.4, 1.2)	20	1.1 (0.3, 1.9)	88	4.2 (2.9, 5.5)
aPD (95% CI; q-value)	Referent		0.8 (-0.3, 1.9; 0.19)		4.3 (2.5, 6.1; <0.001 )	

Adjusted prevalence differences (aPDs) are absolute percentage point (pp) differences for “very or extremely likely” responses. Models are adjusted for age (continuous), race and ethnicity (7 categories: white, non-Hispanic; Black, non-Hispanic; Hispanic, any race; Asian American/Pacific Islander, non-Hispanic; American Indian/Alaska native, non-Hispanic; 2 + races, non-Hispanic; some other race, non-), gender (3 categories: male, female, other), income (7 categories: <$10,000, $10,000-$24,999, $25,000-$49,999, $50,000-$74,999, $75,000-$99,999, $100,000-$149,999, ≥$150,000), education (5 categories: no high school diploma or GED, high school graduate/GED, some college or associate’s degree, bachelor’s degree, master’s degree or higher), Census division (9 categories: New England, Middle Atlantic, South Atlantic, East North Central, East South Central, West North Central, West South Central, Mountain, Pacific), marital status (currently married, 2 categories: no, yes), homeownership (2 categories: no, yes), political ideology (conservative, 2 categories: no, yes), rurality (urban residence, 2 categories: no, yes), alcohol consumption (drinks/week, 3 categories: 0, 1–10, ≥ 11), military service (2 categories: no, yes), and history of non-traffic arrest (2 categories: no, yes)

For a given value of the false discovery rate, Q, the q-values are chosen so that, of comparisons that reject the null hypothesis (decided by q < α), the expected proportion of these comparisons that incorrectly reject the null hypothesis is at most Q. Item non-responses are not reported in the tables but are included in the prevalence calculations

### Attitudes, beliefs, and behaviors

NRA approvers and non-approvers differed, sometimes widely, on the attitudes, beliefs, and behaviors related to political violence that were part of this analysis.

#### Democracy, authoritarianism, and elections

NRA approvers and non-approvers differed significantly on 12 of 16 measures concerning attitudes toward democracy, authoritarianism, and elections (Table 9). For example, approvers were less likely than non-approvers to report strong or very strong agreement with the statement that “democracy is the best form of government” (approvers 69.1%, 95% CI 66.6%, 71.7%; non-approvers 74.2%, 95% CI 72.1%, 76.2%; aPD - 5.9pp, 95% CI -10.0pp, -1.7pp; q = 0.007) or that it was very or extremely important “for the United States to remain a democracy” (approvers 85.2%, 95% CI 83.0%, 87.3%; non-approvers 91.9%, 95% CI 90.5%, 93.3%; aPD - 6.0pp, 95% CI -9.5pp, -2.5pp; q = 0.001).

**Table 9:** NRA approval and beliefs concerning democracy, authoritarianism, and elections

Statement	How Much Do You Approve or Disapprove of…the National Rifle Association?					
	Do Not Approve		Somewhat Approve		Strongly/Very Strongly Approve	
	Unweighted *n*	Weighted % (95% CI)	Unweighted *n*	Weighted % (95% CI)	Unweighted *n*	Weighted % (95% CI)
Democracy is the best form of government.						
Do not agree	109	4.6 (3.5, 5.8)	102	5.6 (4.2, 6.9)	214	10.3 (8.5, 12.0)
Somewhat agree	569	20.3 (18.4, 22.2)	441	25.1 (22.5, 27.8)	418	18.7 (16.5, 20.9)
Strongly or very strongly agree	2721	74.2 (72.1, 76.2)	1712	68.1 (65.3, 70.8)	2000	69.1 (66.6, 71.7)
aPD (95% CI; q-value)	Referent		-7.1 (-10.8, -3.4; <0.001 )		-5.9 (-10.0, -1.7; 0.007)	
How important do you think it is for the United States to remain a democracy?						
Not important	41	2.0 (1.2, 2.8)	48	3.1 (2.0, 4.2)	99	5.1 (3.8, 6.4)
Somewhat important	128	5.7 (4.5, 6.9)	142	10.2 (8.1, 12.2)	139	8.2 (6.4, 10.0)
Very or extremely important	3242	91.9 (90.5, 93.3)	2065	85.8 (83.5, 88.1)	2397	85.2 (83.0, 87.3)
aPD (95% CI; q-value)	Referent		-5.1 (-8.0, -2.2; <0.001 )		-6.0 (-9.5, -2.5; 0.001)	
When thinking about democracy in the United States these days, do you believe…						
There is a serious threat to our democracy.	2614	70.7 (68.6, 72.8)	1390	56.6 (53.8, 59.4)	1959	69.3 (66.7, 71.8)
There may be a threat to our democracy, but it is not serious.	130	5.2 (4.1, 6.3)	143	6.6 (5.1, 8.1)	160	6.5 (5.1, 8.0)
There is no threat to our democracy.	662	23.4 (21.4, 25.3)	711	35.2 (32.4, 37.9)	518	22.5 (20.2, 24.8)
aPD (95% CI; q-value)	Referent		-16.2 (-20.1, -12.3; <0.001 )		-3.7 (-8.0, 0.6; 0.1)	
How much is each of the following a threat to democracy in the United States?						
The influence of big money in elections						
A serious threat	2739	75.6 (73.6, 77.6)	1598	67.0 (64.2, 69.7)	1956	69.6 (67.0, 72.2)
A threat, but not a serious threat	605	20.8 (18.9, 22.7)	572	27.6 (25.0, 30.2)	599	25.3 (22.9, 27.8)
Not a threat	59	2.7 (1.8, 3.5)	83	4.4 (3.1, 5.7)	95	3.8 (2.7, 4.8)
aPD (95% CI; q-value)	Referent		-8.6 (-12.4, -4.9; <0.001 )		-6.9 (-11.2, -2.6; 0.002)	
The influence of foreign governments in elections						
A serious threat	1944	51.9 (49.7, 54.1)	1106	45.7 (42.9, 48.5)	1529	54.8 (52.1, 57.5)
A threat, but not a serious threat	1288	39.9 (37.7, 42.1)	959	44.0 (41.2, 46.8)	883	33.8 (31.3, 36.4)
Not a threat	175	7.5 (6.2, 8.9)	187	9.4 (7.6, 11.1)	234	9.8 (8.0, 11.5)
aPD (95% CI; q-value)	Referent		-7.3 (-11.2, -3.4; <0.001 )		0.4 (-3.9, 4.8; 0.85)	
The possibility of political violence						
A serious threat	2378	65.4 (63.2, 67.6)	1075	47.7 (44.9, 50.5)	1161	44.2 (41.6, 46.8)
A threat, but not a serious threat	929	29.5 (27.5, 31.6)	1011	42.9 (40.1, 45.7)	1201	43.5 (40.9, 46.1)
Not a threat	98	4.3 (3.3, 5.4)	166	8.3 (6.6, 10.1)	282	10.8 (9.1, 12.5)
aPD (95% CI; q-value)	Referent		-15.0 (-18.9, -11.2; <0.001 )		-17.6 (-21.9, -13.3; <0.001 )	
The influence of white nationalist groups						
A serious threat	2598	71.9 (69.9, 74.0)	878	39.4 (36.6, 42.1)	572	22.7 (20.4, 24.9)
A threat, but not a serious threat	664	22.0 (20.1, 23.9)	879	37.5 (34.8, 40.2)	920	34.7 (32.2, 37.2)
Not a threat	141	5.1 (4.0, 6.2)	490	21.8 (19.4, 24.1)	1150	41.0 (38.5, 43.6)
aPD (95% CI; q-value)	Referent		-25.5 (-29.3, -21.8; <0.001 )		-37.3 (-41.3, -33.3; <0.001 )	
The influence of groups like Black Lives Matter						
A serious threat	277	8.6 (7.4, 9.9)	658	27.0 (24.6, 29.4)	1565	55.4 (52.8, 58.1)
A threat, but not a serious threat	744	22.1 (20.2, 23.9)	873	38.2 (35.5, 41.0)	745	28.7 (26.3, 31.2)
Not a threat	2382	68.5 (66.4, 70.6)	721	33.8 (31.1, 36.4)	342	14.9 (12.9, 16.8)
aPD (95% CI; q-value)	Referent		10.5 (7.6, 13.4; <0.001 )		31.9 (28.2, 35.5; <0.001 )	
Efforts to overturn the results of elections						
A serious threat	2755	74.3 (72.2, 76.4)	1114	46.6 (43.9, 49.4)	1076	41.6 (39.0, 44.2)
A threat, but not a serious threat	505	18.6 (16.7, 20.4)	837	38.5 (35.7, 41.3)	930	34.2 (31.7, 36.7)
Not a threat	146	6.3 (5.1, 7.6)	301	13.8 (11.9, 15.8)	640	23.1 (20.9, 25.3)
aPD (95% CI; q-value)	Referent		-22.7 (-26.5, -18.9; <0.001 )		-25.6 (-29.9, -21.3; <0.001 )	
These days, American democracy only serves the interests of the wealthy and powerful.						
Do not agree	947	24.1 (22.3, 25.9)	722	29.0 (26.5, 31.5)	881	29.0 (26.7, 31.2)
Somewhat agree	1432	41.8 (39.6, 44.0)	963	43.3 (40.5, 46.1)	873	33.1 (30.7, 35.6)
Strongly or very strongly agree	1031	33.6 (31.4, 35.7)	568	26.3 (23.9, 28.8)	879	35.9 (33.4, 38.5)
aPD (95% CI; q-value)	Referent		-6.1 (-9.9, -2.4; 0.002)		2.5 (-1.8, 6.8; 0.27)	
Having a strong leader for America is more important than having a democracy.						
Do not agree	2704	73.6 (71.5, 75.7)	1498	58.7 (55.9, 61.6)	1547	51.6 (48.9, 54.2)
Somewhat agree	402	14.7 (12.9, 16.4)	459	24.7 (22, 27.3)	523	22.9 (20.6, 25.3)
Strongly or very strongly agree	298	10.9 (9.4, 12.4)	291	15.0 (13.0, 17.1)	562	23.6 (21.3, 25.9)
aPD (95% CI; q-value)	Referent		3.9 (0.9, 6.9; 0.02)		10.5 (6.8, 14.2; <0.001 )	
We should suspend Congress for a few years so a strong leader can clean up the mess made by politicians in Washington.						
Do not agree	2677	73.2 (71.2, 75.3)	1530	61.2 (58.3, 64.0)	1534	50.5 (47.8, 53.1)
Somewhat agree	429	15.5 (13.7, 17.2)	455	23.5 (20.9, 26.1)	522	23.2 (20.9, 25.5)
Strongly or very strongly agree	293	10.1 (8.7, 11.5)	269	14.0 (11.9, 16.0)	585	24.8 (22.4, 27.2)
aPD (95% CI; q-value)	Referent		3.1 (0.1, 6.1; 0.05)		11.8 (8.1, 15.6; <0.001 )	
I can trust the American people as a whole when it comes to making judgments under our democratic system about the issues facing our country.						
Do not agree	720	23.7 (21.7, 25.7)	552	25.1 (22.7, 27.5)	810	32.0 (29.5, 34.5)
Somewhat agree	1888	55.2 (53, 57.4)	1231	54.3 (51.5, 57.1)	1190	44.4 (41.8, 47.0)
Strongly or very strongly agree	797	20.4 (18.7, 22.1)	473	19.3 (17.2, 21.5)	646	22.6 (20.4, 24.8)
aPD (95% CI; q-value)	Referent		-1.0 (-4.2, 2.1; 0.52)		1.9 (-1.7, 5.6; 0.31)	
The 2020 election was stolen from Donald Trump, and Joe Biden is an illegitimate president.						
Do not agree	3226	91.8 (90.4, 93.2)	1541	66.9 (64.2, 69.6)	732	28.3 (25.9, 30.7)
Somewhat agree	79	3.6 (2.7, 4.6)	409	18.4 (16.2, 20.7)	714	25.5 (23.2, 27.8)
Strongly or very strongly agree	105	4.1 (3.1, 5.1)	288	12.7 (10.9, 14.6)	1185	44.4 (41.8, 47)
aPD (95% CI; q-value)	Referent		2.5 (0, 5.1; 0.06)		30.0 (26.3, 33.6; <0.001 )	
Armed citizens should patrol polling places at election time. (Asked in 2022)						
Do not agree	3274	93.9 (92.6, 95.1)	2027	85.3 (82.9, 87.6)	1988	70.2 (67.6, 72.7)
Somewhat agree	91	4.0 (3.0, 5.0)	170	10.5 (8.5, 12.6)	366	15.9 (13.9, 18.0)
Strongly or very strongly agree	47	2.1 (1.4, 2.8)	62	4.2 (2.8, 5.5)	296	13.9 (11.9, 15.9)
aPD (95% CI; q-value)	Referent		2.3 (0.3, 4.2; 0.03)		11.7 (8.9, 14.5; <0.001 )	
Which is more important to you?						
Having election outcomes determined democratically	2649	72.0 (69.9, 74.2)	1385	55.7 (52.9, 58.6)	1449	49.7 (47.1, 52.3)
OR						
Having political leaders I can trust to look out for my values and interests	690	24.3 (22.3, 26.3)	830	39.8 (37.0, 42.5)	1156	46.6 (43.9, 49.2)
aPD (95% CI; q-value)	Referent		12.0 (8.2, 15.8; <0.001 )		15.2 (10.8, 19.5; <0.001 )	

Adjusted prevalence (aPDs) are absolute percentage point (pp) differences for the following responses: item 1, “strongly or very strongly agree”; item 2, “very or extremely important”; items 3–9, “a serious threat”; items 10–15, “strongly or very strongly agree”; item 16, “having political leaders I can trust to look out for my values and interests.” Models are adjusted for age (continuous), race and ethnicity (7 categories: white, non-Hispanic; Black, non-Hispanic; Hispanic, any race; Asian American/Pacific Islander, non-Hispanic; American Indian/Alaska native, non-Hispanic; 2 + races, non-Hispanic; some other race, non-), gender (3 categories: male, female, other), income (7 categories: <$10,000, $10,000-$24,999, $25,000-$49,999, $50,000-$74,999, $75,000-$99,999, $100,000-$149,999, ≥$150,000), education (5 categories: no high school diploma or GED, high school graduate/GED, some college or associate’s degree, bachelor’s degree, master’s degree or higher), Census division (9 categories: New England, Middle Atlantic, South Atlantic, East North Central, East South Central, West North Central, West South Central, Mountain, Pacific), marital status (currently married, 2 categories: no, yes), homeownership (2 categories: no, yes), political ideology (conservative, 2 categories: no, yes), rurality (urban residence, 2 categories: no, yes), alcohol consumption (drinks/week, 3 categories: 0, 1–10, ≥ 11), military service (2 categories: no, yes), and history of non-traffic arrest (2 categories: no, yes)

For a given value of the false discovery rate, Q, the q-values are chosen so that, of comparisons that reject the null hypothesis (decided by q < α), the expected proportion of these comparisons that incorrectly reject the null hypothesis is at most Q. Item non-responses are not reported in the tables but are included in the prevalence calculations

Approvers were more likely than non-approvers to report strong or very strong agreement with statements endorsing authoritarianism, such as “having a strong leader for America is more important than having a democracy” (approvers 23.6%, 95% CI 21.3%, 25.9%; non-approvers 10.9%, 95% CI 9.4%, 12.4%; aPD 10.5pp, 95% CI 6.8pp, 14.2pp q < 0.001) and “we should suspend Congress for a few years so a strong leader can clean up the mess made by politicians in Washington” (approvers 24.8%, 95% CI 22.4%, 27.2%; non-approvers 10.1%, 95% CI 8.7%, 11.5%; aPD 11.8pp, 95% CI 8.1pp,15.6pp; q < 0.001). They also more frequently agreed strongly or very strongly that “armed citizens should patrol polling places at election time” (approvers 13.9%, 95% CI 11.9%, 15.9%; non-approvers 2.1%, 95% CI 1.4%, 2.8%; aPD 11.7pp, 95% CI 8.9pp, 14.5pp; q < 0.001).

#### Party affiliation, political ideology, and partisanship

NRA approvers were more likely than non-approvers to identify as Republicans, and particularly as strong Republicans (approvers 40.5%, 95% CI 37.9%, 43.0%; non-approvers 1.9%, 95% CI 1.3%, 2.5%; aPD 37.4pp, 95% CI 33.9pp, 41.0pp; q < 0.001) (Table 10). They also more commonly identified as Make America Great Again (MAGA) Republicans (approvers 32.4%, 95% CI 30.0%, 34.8%; non-approvers 0.9%, 95% CI 0.5%, 1.4%; aPD 34.9pp, 95% CI 31.5pp, 38.4pp; q < 0.001) and as extreme conservatives (approvers 12.8%, 95% CI 11.1%, 14.5%; non-approvers 1.0%, 95% CI 0.6%, 1.5%; aPD 10.8pp, 95% CI 9.0pp, 12.6pp; q < 0.001). Among Republicans, NRA approvers were more likely than non-approvers to see Democrats as “enemies – that is, if they win, your life or your entire way of life may be threatened.” Among Democrats, NRA approvers were less likely than non-approvers to see Republicans as enemies (Table 10).

**Table 10:** NRA approval, party affiliation, political ideology, and partisanship

Condition	How Much Do You Approve or Disapprove of…the National Rifle Association?					
	Do Not Approve		Somewhat Approve		Strongly/Very Strongly Approve	
	Unweighted *n*	Weighted % (95% CI)	Unweighted *n*	Weighted % (95% CI)	Unweighted *n*	Weighted % (95% CI)
Party affiliation						
Strong Republican	66	1.9 (1.3, 2.5)	368	13.9 (12.2, 15.7)	1180	40.5 (37.9, 43.0)
Not very strong Republican	111	3.7 (2.8, 4.7)	435	18.2 (16.2, 20.3)	454	17.9 (15.8, 19.9)
Leans Republican	110	3.4 (2.6, 4.2)	386	14.9 (13, 16.8)	534	17.3 (15.5, 19.2)
Middle of the road	410	13.4 (11.8, 15.0)	401	20.1 (17.8, 22.5)	313	14.4 (12.4, 16.4)
Leans Democrat	781	21.3 (19.5, 23.1)	230	9.4 (7.8, 11.0)	38	1.9 (1.1, 2.6)
Not very strong Democrat	607	20.1 (18.3, 22.0)	247	12.8 (10.9, 14.8)	90	4.7 (3.3, 6.1)
Strong Democrat	1333	36.2 (34.1, 38.3)	200	10.6 (8.7, 12.5)	56	3.3 (2.1, 4.6)
aPD (95% CI; q-value)	Referent		21.6 (18.6, 24.6; <0.001 )		37.4 (33.9, 41.0; <0.001 )	
MAGA identification						
MAGA Republican	27	0.9 (0.5, 1.4)	193	7.2 (5.9, 8.5)	915	32.4 (30.0, 34.8)
MAGA supporter, Republican	16	0.5 (0.2, 0.8)	177	6.8 (5.6, 8.1)	430	14.0 (12.3, 15.7)
MAGA supporter, Non-Republican	31	1.3 (0.7, 1.9)	54	3.4 (2.1, 4.7)	108	4.5 (3.2, 5.7)
Non-MAGA Republican	242	7.6 (6.4, 8.8)	789	33.4 (30.8, 36.1)	786	29.9 (27.5, 32.4)
Non-MAGA, Non-Republican	3064	89.7 (88.3, 91.1)	982	49.1 (46.3, 52.0)	370	19.2 (16.8, 21.5)
aPD (95% CI; q-value)	Referent		6.5 (4.1, 8.8; <0.001 )		34.9 (31.5, 38.4; <0.001 )	
Political ideology						
Extremely liberal	299	9.6 (8.3, 10.9)	25	1.4 (0.7, 2.1)	24	1.5 (0.7, 2.3)
Liberal	1002	28.7 (26.7, 30.7)	136	6.6 (5.2, 8.0)	43	2.4 (1.4, 3.4)
Slightly liberal	573	16.5 (14.9, 18.2)	138	7.5 (5.8, 9.1)	47	2.2 (1.4, 3)
Moderate/middle of the road	1124	33.8 (31.7, 36.0)	812	40.2 (37.4, 43.0)	513	24.4 (22.0, 26.8)
Slightly conservative	211	6.3 (5.1, 7.5)	450	18.5 (16.4, 20.6)	404	15.7 (13.6, 17.7)
Conservative	126	4.0 (3.1, 4.9)	589	22.4 (20.2, 24.7)	1249	41.0 (38.5, 43.5)
Extremely conservative	31	1.0 (0.6, 1.5)	81	3.4 (2.4, 4.4)	353	12.8 (11.1, 14.5)
aPD (95% CI; q-value)	Referent		1.7 (0.6, 2.8 ; 0.002)		10.8 (9.0, 12.6 ; <0.001 )	
Partisanship						
Asked of Republicans						
Democrats are political opposition	224	76.4 (69.8, 83.1)	829	70.1 (66.6, 73.5)	1046	49.3 (46.4, 52.2)
Democrats are enemies	57	21.4 (14.9, 27.8)	335	27.5 (24.1, 30.9)	1103	49.3 (46.5, 52.2)
aPD (95% CI; q-value)	Referent		3.4 (-4.0, 10.8; 0.37)		21.6 (14.0, 29.2; <0.001 )	
Asked of Democrats						
Republicans are political opposition	1462	53.0 (50.6, 55.5)	490	68.7 (63.5, 73.8)	140	72.1 (62.1, 82.0)
Republicans are enemies	1240	46.2 (43.7, 48.7)	180	30.1 (25.1, 35.2)	40	23.1 (14.3, 31.9)
aPD (95% CI; q-value)	Referent		-18.4 (-23.7, -13.0; <0.001 )		-25.9 (-36.3, -15.5; <0.001 )	

Adjusted prevalence (aPDs) are absolute percentage point (pp) differences for the following responses: party affiliation, “Strong Republican”; MAGA identification, “MAGA Republican”; political ideology, “Extremely conservative”; partisanship, “Enemies.” Models are adjusted for age (continuous), race and ethnicity (7 categories: white, non-Hispanic; Black, non-Hispanic; Hispanic, any race; Asian American/Pacific Islander, non-Hispanic; American Indian/Alaska native, non-Hispanic; 2 + races, non-Hispanic; some other race, non-), gender (3 categories: male, female, other), income (7 categories: <$10,000, $10,000-$24,999, $25,000-$49,999, $50,000-$74,999, $75,000-$99,999, $100,000-$149,999, ≥$150,000), education (5 categories: no high school diploma or GED, high school graduate/GED, some college or associate’s degree, bachelor’s degree, master’s degree or higher), Census division (9 categories: New England, Middle Atlantic, South Atlantic, East North Central, East South Central, West North Central, West South Central, Mountain, Pacific), marital status (currently married, 2 categories: no, yes), homeownership (2 categories: no, yes), political ideology (conservative, 2 categories: no, yes), rurality (urban residence, 2 categories: no, yes), alcohol consumption (drinks/week, 3 categories: 0, 1–10, ≥ 11), military service (2 categories: no, yes), and history of non-traffic arrest (2 categories: no, yes)

For a given value of the false discovery rate, Q, the q-values are chosen so that, of comparisons that reject the null hypothesis (decided by q < α), the expected proportion of these comparisons that incorrectly reject the null hypothesis is at most Q. Item non-responses are not reported in the tables but are included in the prevalence calculations

#### Hatred, fear, and enmity toward others

Prevalences of strong agreement on scales assessing homonegativity, racism, transphobia, xenophobia, hostile sexism, Islamophobia, and antisemitism were higher among NRA approvers than among non-approvers (Table 11). Prevalences among approvers ranged from 56.3% (95% CI 55.2%, 57.5) for homonegativity to 4.0% (95% CI 3.5%, 4.5%) for antisemitism, and aPDs ranged from 34.7pp (95% CI 31.3pp, 38.1pp; q < 0.001) for racism to 2.6pp (95% CI 0.8pp, 4.5pp; q = 0.008) for antisemitism.

**Table 11:** NRA approval and 7 forms of hatred, fear, and enmity toward others

Condition	How Much Do You Approve or Disapprove of…the National Rifle Association?					
	Do Not Approve		Somewhat Approve		Strongly/Very Strongly Approve	
	Unweighted *n*	Weighted % (95% CI)	Unweighted *n*	Weighted % (95% CI)	Unweighted *n*	Weighted % (95% CI)
Homonegativity						
Non-agreement	1038	29.0 (28.1, 29.9)	153	7.5 (6.9, 8.2)	38	1.8 (1.5, 2.2)
Weak Agreement	1568	43.8 (42.9, 44.8)	642	31.6 (30.4, 32.8)	250	12.1 (11.3, 12.9)
Moderate agreement	635	17.8 (17.0, 18.6)	666	32.8 (31.6, 34.0)	616	29.8 (28.6, 30.9)
Strong agreement	335	9.4 (8.8, 10.0)	571	28.1 (27.0, 29.2)	1167	56.3 (55.2, 57.5)
aPD (95% CI; q-value)	Referent		10.5 (7.5, 13.5; <0.001 )		31.6 (27.8, 35.3; <0.001 )	
Racism						
Non-agreement	1403	39.2 (38.2, 40.2)	192	9.4 (8.7, 10.2)	44	2.1 (1.8, 2.5)
Weak Agreement	1275	35.7 (34.7, 36.6)	482	23.7 (22.6, 24.8)	157	7.6 (6.9, 8.3)
Moderate agreement	761	21.3 (20.4, 22.1)	944	46.5 (45.2, 47.8)	786	37.9 (36.8, 39.1)
Strong agreement	138	3.8 (3.5, 4.2)	413	20.3 (19.4, 21.3)	1084	52.3 (51.2, 53.5)
aPD (95% CI; q-value)	Referent		9.2 (6.8, 11.6; <0.001 )		34.7 (31.3, 38.1; <0.001 )	
Transphobia						
Non-agreement	896	25.1 (24.2, 25.9)	132	6.5 (5.8, 7.2)	68	3.3 (2.8, 3.7)
Weak Agreement	1734	48.5 (47.5, 49.5)	764	37.6 (36.4, 38.8)	416	20.1 (19.1, 21.0)
Moderate agreement	690	19.3 (18.5, 20.1)	793	39.1 (37.8, 40.3)	888	42.9 (41.7, 44.1)
Strong agreement	256	7.2 (6.7, 7.7)	342	16.8 (15.9, 17.8)	699	33.8 (32.7, 34.9)
aPD (95% CI; q-value)	Referent		5.7 (3.0, 8.5; <0.001 )		19.5 (16.0, 22.9; <0.001 )	
Xenophobia						
Non-agreement	1563	43.7 (42.7, 44.7)	323	15.9 (15.0, 16.8)	152	7.3 (6.6, 8.1)
Weak Agreement	1576	44.1 (43.1, 45.0)	993	48.9 (47.7, 50.2)	690	33.3 (32.2, 34.4)
Moderate agreement	321	9.0 (8.3, 9.6)	523	25.8 (24.6, 26.9)	753	36.4 (35.2, 37.5)
Strong agreement	117	3.3 (2.9, 3.6)	191	9.4 (8.7, 10.2)	476	23.0 (22.0, 23.9)
aPD (95% CI; q-value)	Referent		3.7 (1.3, 6.0; 0.003)		14.8 (11.9, 17.6; <0.001 )	
Hostile Sexism						
Non-agreement	1957	54.7 (53.7, 55.7)	600	29.5 (28.4, 30.7)	346	16.7 (15.8, 17.6)
Weak Agreement	1176	32.9 (32.0, 33.8)	882	43.4 (42.2, 44.6)	914	44.1 (43.0, 45.3)
Moderate agreement	315	8.8 (8.2, 9.4)	406	20.0 (18.9, 21.1)	518	25.0 (24.0, 26.1)
Strong agreement	128	3.6 (3.2, 4.0)	144	7.1 (6.3, 7.8)	293	14.1 (13.2, 15.0)
aPD (95% CI; q-value)	Referent		3.0 (0.7, 5.4; 0.01)		9.6 (6.8, 12.4; <0.001 )	
Islamophobia						
Non-agreement	2264	63.3 (62.3, 64.2)	1001	49.3 (48.0, 50.5)	702	33.9 (32.8, 35.0)
Weak Agreement	968	27.1 (26.2, 27.9)	701	34.5 (33.3, 35.7)	688	33.2 (32.1, 34.3)
Moderate agreement	267	7.5 (6.9, 8.0)	255	12.6 (11.7, 13.5)	451	21.8 (20.8, 22.7)
Strong agreement	79	2.2 (1.9, 2.5)	74	3.7 (3.1, 4.2)	230	11.1 (10.4, 11.9)
aPD (95% CI; q-value)	Referent		0.1 (-1.6, 1.9; 0.87)		7.1 (4.7, 9.4; <0.001 )	
Antisemitism						
Non-agreement	2189	61.2 (60.2, 62.2)	997	49.1 (47.8, 50.3)	970	46.8 (45.7, 48.0)
Weak Agreement	823	23.0 (22.2, 23.8)	650	32.0 (30.8, 33.2)	686	33.1 (32.0, 34.2)
Moderate agreement	489	13.7 (12.9, 14.5)	322	15.9 (14.8, 16.9)	333	16.1 (15.1, 17.1)
Strong agreement	76	2.1 (1.9, 2.4)	62	3.1 (2.5, 3.6)	82	4.0 (3.5, 4.5)
aPD (95% CI; q-value)	Referent		1.5 (-0.2, 3.2; 0.1)		2.6 (0.8, 4.5; 0.008)	

Adjusted prevalence differences (aPDs) are absolute percentage point (pp) differences for the “strong agreement” category. Models are adjusted for age (continuous), race and ethnicity (7 categories: white, non-Hispanic; Black, non-Hispanic; Hispanic, any race; Asian American/Pacific Islander, non-Hispanic; American Indian/Alaska native, non-Hispanic; 2 + races, non-Hispanic; some other race, non-), gender (3 categories: male, female, other), income (7 categories: <$10,000, $10,000-$24,999, $25,000-$49,999, $50,000-$74,999, $75,000-$99,999, $100,000-$149,999, ≥$150,000), education (5 categories: no high school diploma or GED, high school graduate/GED, some college or associate’s degree, bachelor’s degree, master’s degree or higher), Census division (9 categories: New England, Middle Atlantic, South Atlantic, East North Central, East South Central, West North Central, West South Central, Mountain, Pacific), marital status (currently married, 2 categories: no, yes), homeownership (2 categories: no, yes), political ideology (conservative, 2 categories: no, yes), rurality (urban residence, 2 categories: no, yes), alcohol consumption (drinks/week, 3 categories: 0, 1–10, ≥ 11), military service (2 categories: no, yes), and history of non-traffic arrest (2 categories: no, yes)

For a given value of the false discovery rate, Q, the q-values are chosen so that, of comparisons that reject the null hypothesis (decided by q < α), the expected proportion of these comparisons that incorrectly reject the null hypothesis is at most Q. Item non-responses are not reported in the tables but are included in the prevalence calculations

#### Conspiracism

NRA approvers were more likely than non-approvers to endorse a wide array of conspiracy theories (Table 12), including that “the government permits or perpetrates acts of terrorism on its own soil, disguising its involvement” (approvers 29.3%, 95% CI 26.9%, 31.7%; non-approvers 7.8%, 95% CI 6.5%, 9.0%; aPD 18.8pp, 95% CI 15.2pp, 22.4pp; q < 0.001) and that “the spread of certain viruses and/or diseases is the result of the deliberate, concealed efforts of some organization” (approvers 33.8%, 95% CI 31.3%, 36.4%; non-approvers 6.2%, 95% CI 5.0%, 7.3%; aPD 22.7pp, 95% CI 19.1pp, 26.2pp; q < 0.001).

**Table 12:** NRA approval and conspiracism

There is often debate about whether the public is told the whole truth about various important issues. How much do you agree or disagree with the following statements?	How Much Do You Approve or Disapprove of…the National Rifle Association?					
	Do Not Approve		Somewhat Approve		Strongly/Very Strongly Approve	
	Unweighted *n*	Weighted % (95% CI)	Unweighted *n*	Weighted % (95% CI)	Unweighted *n*	Weighted % (95% CI)
The government is involved in the murder of innocent citizens and/or well-known public figures and keeps this a secret.						
Do not agree	2571	69.1 (67.0, 71.2)	1359	54.8 (51.9, 57.6)	1094	38.7 (36.2, 41.3)
Somewhat agree	577	20.4 (18.5, 22.3)	651	31.2 (28.5, 33.9)	887	33.0 (30.6, 35.4)
Strongly or very strongly agree	233	9.0 (7.5, 10.4)	228	12.4 (10.4, 14.4)	641	26.1 (23.7, 28.5)
aPD (95% CI; q-value)	Referent		2.4 (-0.4, 5.2; 0.1)		15.2 (11.6, 18.7; <0.001 )	
The spread of certain viruses and/or diseases is the result of the deliberate, concealed efforts of some organization.						
Do not agree	2740	75.3 (73.2, 77.3)	1246	50.4 (47.6, 53.2)	789	27.6 (25.3, 29.9)
Somewhat agree	486	17.4 (15.5, 19.2)	715	32.9 (30.2, 35.7)	1001	36.4 (33.9, 38.9)
Strongly or very strongly agree	164	6.2 (5.0, 7.3)	279	15.0 (12.8, 17.1)	836	33.8 (31.3, 36.4)
aPD (95% CI; q-value)	Referent		5.9 (3.1, 8.7; <0.001 )		22.7 (19.1, 26.2; <0.001 )	
The government permits or perpetrates acts of terrorism on its own soil, disguising its involvement.						
Do not agree	2513	66.9 (64.7, 69.1)	1204	48.1 (45.3, 50.9)	805	29.3 (27.0, 31.7)
Somewhat agree	661	24.1 (22.0, 26.1)	768	35.6 (32.9, 38.4)	1054	38.9 (36.3, 41.4)
Strongly or very strongly agree	217	7.8 (6.5, 9.0)	263	14.4 (12.2, 16.5)	759	29.3 (26.9, 31.7)
aPD (95% CI; q-value)	Referent		4.5 (1.7, 7.3; 0.003)		18.8 (15.2, 22.4; <0.001 )	
Technology with mind-control capacities is used on people without their knowledge.						
Do not agree	2694	76.4 (74.4, 78.4)	1474	61.1 (58.2, 63.9)	1314	45.8 (43.2, 48.4)
Somewhat agree	535	16.5 (14.7, 18.2)	595	27.3 (24.6, 29.9)	859	31.8 (29.3, 34.2)
Strongly or very strongly agree	168	6.0 (4.9, 7.2)	172	10.2 (8.2, 12.2)	450	20.1 (17.9, 22.4)
aPD (95% CI; q-value)	Referent		4.2 (1.3, 7.0; 0.005)		13.7 (10.2, 17.3; <0.001 )	
The government falsely blames innocent people to hide its involvement in criminal activity.						
Do not agree	2310	60.2 (58.0, 62.5)	1031	41.5 (38.8, 44.2)	548	21.0 (18.8, 23.2)
Somewhat agree	803	28.3 (26.2, 30.4)	854	38.8 (36.1, 41.6)	1086	38.7 (36.1, 41.2)
Strongly or very strongly agree	275	10.0 (8.6, 11.5)	356	18.0 (15.7, 20.3)	997	38.0 (35.4, 40.6)
aPD (95% CI; q-value)	Referent		4.5 (1.4, 7.6; 0.005)		22.2 (18.4, 26; <0.001 )	
Experiments involving new drugs or technologies are routinely carried out on the public without their knowledge or consent.						
Do not agree	2513	68.7 (66.5, 70.8)	1280	50.5 (47.7, 53.4)	1016	35.8 (33.3, 38.3)
Somewhat agree	650	21.9 (20.0, 23.8)	725	34.3 (31.6, 37.0)	994	35.3 (32.8, 37.8)
Strongly or very strongly agree	226	8.2 (6.9, 9.6)	238	13.8 (11.7, 16.0)	611	26.6 (24.1, 29.0)
aPD (95% CI; q-value)	Referent		4.3 (1.3, 7.2; 0.005)		16.0 (2.2, 19.7; <0.001 )	

Adjusted prevalence differences (aPDs) are absolute percentage point (pp) differences for “strongly or very strongly agree” responses. Models are adjusted for age (continuous), race and ethnicity (7 categories: white, non-Hispanic; Black, non-Hispanic; Hispanic, any race; Asian American/Pacific Islander, non-Hispanic; American Indian/Alaska native, non-Hispanic; 2 + races, non-Hispanic; some other race, non-), gender (3 categories: male, female, other), income (7 categories: <$10,000, $10,000-$24,999, $25,000-$49,999, $50,000-$74,999, $75,000-$99,999, $100,000-$149,999, ≥$150,000), education (5 categories: no high school diploma or GED, high school graduate/GED, some college or associate’s degree, bachelor’s degree, master’s degree or higher), Census division (9 categories: New England, Middle Atlantic, South Atlantic, East North Central, East South Central, West North Central, West South Central, Mountain, Pacific), marital status (currently married, 2 categories: no, yes), homeownership (2 categories: no, yes), political ideology (conservative, 2 categories: no, yes), rurality (urban residence, 2 categories: no, yes), alcohol consumption (drinks/week, 3 categories: 0, 1–10, ≥ 11), military service (2 categories: no, yes), and history of non-traffic arrest (2 categories: no, yes)

For a given value of the false discovery rate, Q, the q-values are chosen so that, of comparisons that reject the null hypothesis (decided by q < α), the expected proportion of these comparisons that incorrectly reject the null hypothesis is at most Q. Item non-responses are not reported in the tables but are included in the prevalence calculations

#### QAnon and Christian nationalism

NRA approvers were more likely than non-approvers to endorse the central elements of the QAnon mythology, including that “the government, media, and financial worlds in the U.S. are controlled by a group of Satan-worshipping pedophiles who run a global child sex trafficking operation” (approvers 17.7%, 95% CI 15.6%, 19.8%; non-approvers 4.2%, 95% CI 3.2%, 5.3%; aPD 10.4pp, 95% CI 7.2pp, 13.6pp; q < 0.001) (Table 13).

**Table 13:** NRA approval and endorsement of QAnon and Christian nationalism

Statement	How Much Do You Approve or Disapprove of…the National Rifle Association?					
	Do Not Approve		Somewhat Approve		Strongly/Very Strongly Approve	
	Unweighted *n*	Weighted % (95% CI)	Unweighted *n*	Weighted % (95% CI)	Unweighted *n*	Weighted % (95% CI)
**QAnon**						
The government, media, and financial worlds in the U.S. are controlled by a group of Satan-worshipping pedophiles who run a global child sex trafficking operation.						
Do not agree	3106	87.6 (85.9, 89.3)	1870	78.7 (76.1, 81.3)	1692	59.0 (56.4, 61.7)
Somewhat agree	187	8.1 (6.7, 9.6)	267	14.1 (11.9, 16.2)	535	23.3 (20.9, 25.7)
Strongly or very strongly agree	101	4.2 (3.2, 5.3)	99	7.2 (5.4, 9.1)	391	17.7 (15.6, 19.8)
aPD (95% CI; q-value)	Referent		1.5 (-1.1, 4.1; 0.27)		10.4 (7.2, 13.6; <0.001 )	
There is a storm coming soon that will sweep away the elites in power and restore the rightful leaders.						
Do not agree	2952	84.3 (82.6, 86.1)	1704	71.7 (69.0, 74.4)	1458	54.0 (51.3, 56.6)
Somewhat agree	324	11.2 (9.7, 12.7)	437	21.8 (19.3, 24.3)	787	29.7 (27.3, 32.1)
Strongly or very strongly agree	114	4.5 (3.4, 5.5)	99	6.5 (4.8, 8.2)	372	16.3 (14.2, 18.4)
aPD (95% CI; q-value)	Referent		1.7 (-0.6, 4.1; 0.19)		11.0 (8.0, 14.1; <0.001 )	
**Christian Nationalism**						
The U.S. government should declare America a Christian nation.						
Do not agree	2981	86.0 (84.3, 87.6)	1530	66.5 (63.8, 69.3)	1176	44.7 (42.0, 47.3)
Somewhat agree	220	7.5 (6.3, 8.8)	391	19.4 (17.0, 21.8)	551	21.3 (19.1, 23.5)
Strongly or very strongly agree	182	6.5 (5.3, 7.7)	318	14.1 (12.1, 16.1)	895	34.0 (31.5, 36.6)
aPD (95% CI; q-value)	Referent		2.4 (-0.3, 5.1; 0.11)		17.6 (14.1, 21.2; <0.001 )	
U.S. laws should be based on Christian values.						
Do not agree	2578	75.7 (73.8, 77.7)	978	47.5 (44.7, 50.3)	635	27.2 (24.8, 29.6)
Somewhat agree	532	14.8 (13.2, 16.4)	742	31.2 (28.5, 33.8)	795	29.3 (26.9, 31.8)
Strongly or very strongly agree	271	9.5 (8.1, 10.8)	513	21.3 (19.1, 23.6)	1207	43.4 (40.8, 46.0)
aPD (95% CI; q-value)	Referent		4.9 (2.1, 7.7; 0.001)		22.4 (18.7, 26.1; <0.001 )	
If the U.S. moves away from our Christian foundations, we will not have a country anymore.						
Do not agree	2777	80.4 (78.5, 82.3)	1090	50.3 (47.5, 53.1)	611	25.6 (23.2, 27.9)
Somewhat agree	336	10.1 (8.7, 11.6)	675	28.2 (25.7, 30.7)	767	28.0 (25.6, 30.4)
Strongly or very strongly agree	277	9.5 (8.1, 10.8)	477	21.5 (19.1, 23.9)	1256	46.5 (43.8, 49.1)
aPD (95% CI; q-value)	Referent		5.1 (2.1, 8.1; 0.002)		24.0 (20.2, 27.7; <0.001 )	
Being Christian is an important part of being truly American.						
Do not agree	2810	81.4 (79.6, 83.2)	1376	61.0 (58.2, 63.8)	1033	39.6 (37.1, 42.2)
Somewhat agree	312	9.5 (8.2, 10.9)	490	22.0 (19.5, 24.5)	669	24.6 (22.3, 26.9)
Strongly or very strongly agree	267	9.1 (7.7, 10.4)	374	17.0 (14.8, 19.1)	927	35.8 (33.2, 38.3)
aPD (95% CI; q-value)	Referent		2.9 (0.1, 5.8; 0.06)		18.4 (14.8, 22.0; <0.001 )	
God has called Christians to exercise dominion over all areas of American society.						
Do not agree	3068	88.6 (87.0, 90.1)	1757	75.4 (72.8, 78.1)	1709	62.7 (60.1, 65.3)
Somewhat agree	163	5.7 (4.6, 6.8)	277	14.6 (12.4, 16.8)	403	17.1 (15.0, 19.3)
Strongly or very strongly agree	150	5.8 (4.6, 6.9)	190	10.0 (8.0, 11.9)	497	20.2 (18.0, 22.3)
aPD (95% CI; q-value)	Referent		1.6 (-1.0, 4.1; 0.26)		9.5 (6.3, 12.7; <0.001 )	

Adjusted prevalence differences (aPDs) are absolute percentage point (pp) differences for “strongly or very strongly agree” responses. Models are adjusted for age (continuous), race and ethnicity (7 categories: white, non-Hispanic; Black, non-Hispanic; Hispanic, any race; Asian American/Pacific Islander, non-Hispanic; American Indian/Alaska native, non-Hispanic; 2 + races, non-Hispanic; some other race, non-), gender (3 categories: male, female, other), income (7 categories: <$10,000, $10,000-$24,999, $25,000-$49,999, $50,000-$74,999, $75,000-$99,999, $100,000-$149,999, ≥$150,000), education (5 categories: no high school diploma or GED, high school graduate/GED, some college or associate’s degree, bachelor’s degree, master’s degree or higher), Census division (9 categories: New England, Middle Atlantic, South Atlantic, East North Central, East South Central, West North Central, West South Central, Mountain, Pacific), marital status (currently married, 2 categories: no, yes), homeownership (2 categories: no, yes), political ideology (conservative, 2 categories: no, yes), rurality (urban residence, 2 categories: no, yes), alcohol consumption (drinks/week, 3 categories: 0, 1–10, ≥ 11), military service (2 categories: no, yes), and history of non-traffic arrest (2 categories: no, yes)

For a given value of the false discovery rate, Q, the q-values are chosen so that, of comparisons that reject the null hypothesis (decided by q < α), the expected proportion of these comparisons that incorrectly reject the null hypothesis is at most Q. Item non-responses are not reported in the tables but are included in the prevalence calculations

They were also more likely to agree strongly or very strongly with statements reflecting Christian nationalist beliefs (Table 13), such as “the U.S. government should declare America a Christian nation” (approvers 34.0%, 95% CI 31.5%, 36.6%; non-approvers 6.5%, 95% CI 5.3%, 7.7%; aPD 17.6pp, 95% CI 14.1pp, 21.2pp; q < 0.001) and “if the U.S. moves away from our Christian foundations, we will not have a country anymore” (approvers 46.5%, 95% CI 43.8%, 49.1%; non-approvers 9.5%, 95% CI 8.1%, 10.8%; aPD 24.0pp, 95% CI 20.2pp, 27.7pp; q < 0.001).

#### Extremist organizations and social movements

NRA approvers were more likely than non-approvers to approve strongly or very strongly of an array of right-wing extremist organizations and social movements (Table 14): the Proud Boys, Oath Keepers, Three Percenters, white supremacy movement, militia movement, and boogaloo movement. Differences were largest for strong or very strong approval of the Oath Keepers (approvers 25.5%, 95% CI 20.7%, 30.3%; non-approvers 0.9%, 95% CI 0.3%, 1.5%; aPD 25.0pp, 95% CI 19.9pp, 30.1pp; q < 0.001).

**Table 14:** NRA approval and endorsement of extremist organizations and social movements

How much do you approve or disapprove of these organizations and social movements?	How Much Do You Approve or Disapprove of…the National Rifle Association?					
	Do Not Approve		Somewhat Approve		Strongly/Very Strongly Approve	
	Unweighted *n*	Weighted % (95% CI)	Unweighted *n*	Weighted % (95% CI)	Unweighted *n*	Weighted % (95% CI)
Proud Boys						
Do not approve	3153	98.8 (98.3, 99.4)	1316	86.7 (83.8, 89.6)	832	64.0 (59.8, 68.1)
Somewhat approve	19	1.0 (0.4, 1.5)	109	10.5 (7.9, 13.1)	239	21.4 (17.8, 25.0)
Strongly or very strongly approve	5	0.2 (0.0, 0.4)	20	2.8 (1.3, 4.3)	122	14.7 (11.4, 18.0)
aPD (95% CI; q-value)	Referent		3.5 (1.6, 5.3; <0.001 )		16.2 (12.3, 20.2; <0.001 )	
Oath Keepers						
Do not approve	2681	98.2 (97.5, 98.9)	879	79.4 (75.3, 83.4)	422	48.6 (43.5, 53.7)
Somewhat approve	23	0.9 (0.5, 1.4)	116	15.2 (11.7, 18.8)	219	25.9 (21.4, 30.4)
Strongly or very strongly approve	18	0.9 (0.3, 1.5)	35	5.4 (3.0, 7.9)	156	25.5 (20.7, 30.3)
aPD (95% CI; q-value)	Referent		4.5 (1.9, 7.1; 0.001)		25.0 (19.9, 30.1; <0.001 )	
Three Percenters						
Do not approve	1841	97.3 (96.2, 98.4)	618	79.6 (74.5, 84.7)	372	60.1 (53.7, 66.4)
Somewhat approve	27	1.9 (0.9, 2.8)	59	14.7 (10.2, 19.2)	103	21.1 (15.9, 26.2)
Strongly or very strongly approve	11	0.9 (0.3, 1.5)	20	5.7 (2.5, 8.8)	67	18.9 (13.2, 24.6)
aPD (95% CI; q-value)	Referent		4.7 (1.5, 8.0; 0.006)		19.0 (13.1, 24.9; <0.001 )	
Qanon						
Do not approve	3031	99.3 (98.9, 99.7)	1256	89.5 (86.6, 92.4)	887	75.1 (71.0, 79.2)
Somewhat approve	11	0.4 (0.1, 0.6)	71	8.6 (5.9, 11.3)	121	12.9 (10.1, 15.7)
Strongly or very strongly approve	7	0.4 (0.0, 0.7)	14	1.9 (0.5, 3.2)	71	12.0 (8.4, 15.6)
aPD (95% CI; q-value)	Referent		2.5 (0.7, 4.4; 0.01)		13.1 (9.2, 17.0; <0.001 )	
Christian nationalist movement						
Do not approve	2432	92.8 (91.3, 94.3)	763	67.8 (63.5, 72.1)	403	39.5 (35.0, 44.0)
Somewhat approve	102	4.8 (3.6, 6.0)	208	22.9 (19.1, 26.6)	295	30.9 (26.5, 35.3)
Strongly or very strongly approve	34	2.4 (1.4, 3.3)	65	9.3 (6.1, 12.5)	239	29.6 (25.2, 34.0)
aPD (95% CI; q-value)	Referent		3.9 (0.7, 7.0; 0.02)		22.5 (17.2, 27.8; <0.001 )	
White supremacy movement						
Do not approve	3303	99.3 (98.9, 99.8)	1893	95 (93.3, 96.7)	1936	91.6 (89.5, 93.7)
Somewhat approve	12	0.6 (0.1, 1.0)	45	4.3 (2.7, 5.9)	70	4.0 (2.7, 5.3)
Strongly or very strongly approve	4	0.1 (0.0, 0.2)	8	0.7 (0.1, 1.3)	56	4.4 (2.7, 6.1)
aPD (95% CI; q-value)	Referent		1.3 (0.3, 2.3; 0.01)		5.5 (3.2, 7.7; <0.001 )	
Militia movement						
Do not approve	2694	96.9 (95.9, 97.9)	1143	84.4 (81.1, 87.6)	833	65.1 (61.2, 69.1)
Somewhat approve	38	2.4 (1.5, 3.4)	133	13.3 (10.2, 16.4)	284	21.3 (18.1, 24.4)
Strongly or very strongly approve	14	0.7 (0.3, 1.1)	16	2.4 (0.9, 3.8)	120	13.6 (10.3, 16.9)
aPD (95% CI; q-value)	Referent		2.1 (0.3, 4.0; 0.03)		14.1 (10.1, 18.0; <0.001 )	
Boogaloo movement						
Do not approve	1946	97.8 (96.7, 98.9)	647	88.1 (83.5, 92.7)	434	78.9 (72.8, 85.0)
Somewhat approve	16	1.5 (0.5, 2.6)	38	9.8 (5.8, 13.9)	29	7.6 (4.4, 10.8)
Strongly or very strongly approve	8	0.7 (0.1, 1.2)	5	2.0 (0.0, 4.6)	28	13.5 (7.8, 19.2)
aPD (95% CI; q-value)	Referent		1.5 (-0.6, 3.6; 0.16)		14.3 (8.2, 20.5; <0.001 )	
Black Lives Matter						
Do not approve	617	20.1 (18.3, 22.0)	1129	45.3 (42.5, 48.1)	2083	76.8 (74.2, 79.3)
Somewhat approve	1166	32.6 (30.5, 34.7)	675	34.7 (31.9, 37.5)	272	12.7 (10.8, 14.6)
Strongly or very strongly approve	1493	47.3 (45.0, 49.5)	320	20.1 (17.4, 22.7)	167	10.5 (8.4, 12.5)
aPD (95% CI; q-value)	Referent		-18.7 (-22.3, -15.1; <0.001 )		-20.4 (-24.2, -16.7; <0.001 )	
Redneck Revolt						
Do not approve	2000	96.9 (95.6, 98.2)	836	88.1 (84.4, 91.8)	574	70.7 (65.3, 76.1)
Somewhat approve	22	1.8 (0.9, 2.8)	49	9.9 (6.5, 13.2)	88	12.7 (8.9, 16.5)
Strongly or very strongly approve	14	1.3 (0.4, 2.1)	11	2.1 (0.3, 3.8)	74	16.6 (11.7, 21.5)
aPD (95% CI; q-value)	Referent		1.9 (-0.5, 4.3; 0.14)		16.4 (11.4, 21.4; <0.001 )	
Antifascist (Antifa) movement						
Do not approve	1891	70.4 (68.1, 72.7)	1488	89.6 (87.1, 92)	2026	92.7 (90.5, 94.9)
Somewhat approve	475	17.9 (16.0, 19.8)	90	7.4 (5.4, 9.4)	30	2.7 (1.4, 4.1)
Strongly or very strongly approve	280	11.7 (10.1, 13.4)	28	3.0 (1.5, 4.6)	37	4.6 (2.7, 6.4)
aPD (95% CI; q-value)	Referent		-5.7 (-8.1, -3.2; <0.001 )		-1.9 (-5.1, 1.3; 0.26)	
Anarchist movement						
Do not approve	2410	91.5 (90.0, 93.1)	1286	90.9 (88.3, 93.5)	1338	89.3 (86.1, 92.4)
Somewhat approve	117	6.1 (4.8, 7.4)	64	7.4 (5.0, 9.7)	40	5.1 (2.9, 7.4)
Strongly or very strongly approve	43	2.4 (1.5, 3.3)	12	1.7 (0.5, 3.0)	29	5.6 (3.2, 8.0)
aPD (95% CI; q-value)	Referent		0.6 (-1.4, 2.5; 0.57)		5.8 (2.2, 9.4; 0.003)	

Adjusted prevalence differences (aPDs) are absolute percentage point (pp) differences for “strongly or very strongly approve” responses. Models are adjusted for age (continuous), race and ethnicity (7 categories: white, non-Hispanic; Black, non-Hispanic; Hispanic, any race; Asian American/Pacific Islander, non-Hispanic; American Indian/Alaska native, non-Hispanic; 2 + races, non-Hispanic; some other race, non-), gender (3 categories: male, female, other), income (7 categories: <$10,000, $10,000-$24,999, $25,000-$49,999, $50,000-$74,999, $75,000-$99,999, $100,000-$149,999, ≥$150,000), education (5 categories: no high school diploma or GED, high school graduate/GED, some college or associate’s degree, bachelor’s degree, master’s degree or higher), Census division (9 categories: New England, Middle Atlantic, South Atlantic, East North Central, East South Central, West North Central, West South Central, Mountain, Pacific), marital status (currently married, 2 categories: no, yes), homeownership (2 categories: no, yes), political ideology (conservative, 2 categories: no, yes), rurality (urban residence, 2 categories: no, yes), alcohol consumption (drinks/week, 3 categories: 0, 1–10, ≥ 11), military service (2 categories: no, yes), and history of non-traffic arrest (2 categories: no, yes)

For a given value of the false discovery rate, Q, the q-values are chosen so that, of comparisons that reject the null hypothesis (decided by q < α), the expected proportion of these comparisons that incorrectly reject the null hypothesis is at most Q. Item non-responses are not reported in the tables but are included in the prevalence calculations

NRA approvers were less likely than non-approvers to approve strongly or very strongly of the left-wing organization Black Lives Matter (approvers 10.5%, 95% CI 8.4%, 12.5%; non-approvers 47.3%, 95% CI 45.0%, 49.5%; aPD -20.4pp, 95% CI -24.2pp, -16.7pp; q < 0.001) but more likely to approve of Redneck Revolt (approvers 16.6%, 95% CI 11.7%, 21.5%; non-approvers 1.3%, 95% CI 0.4%, 2.1%; aPD 16.4pp, 95% CI 11.4pp, 21.4pp; q < 0.001) and of the anarchist movement (approvers 5.6%, 95% CI 3.2%, 8.0%; non-approvers 2.4%, 95% CI 1.5%, 3.3%; aPD 5.8pp, 95% CI 2.2pp, 9.4pp; q = 0.003).

#### Trait aggression and non-political violence

NRA approvers were more likely than non-approvers to agree strongly or very strongly with all statements expressing trait aggression (Table 15), including both measures of physical aggression, such as “given enough provocation, I may hit another person” (approvers 14.5%, 95% CI 12.6%, 16.5%; non-approvers 6.5%, 95% CI 5.3%, 7.6%; aPD 8.1pp, 95% CI 5.1pp, 11.1pp; q < 0.001) and measures of grievance and hostility, such as “other people always seem to get the breaks” (approvers 12.3%, 95% CI 10.3%, 14.3%; non-approvers 6.2%, 95% CI 5.1%, 7.3%; aPD 6.9pp, 95% CI 3.8pp, 10.0pp; q < 0.001).

**Table 15:** NRA approval and trait aggression

Sometimes disagreements and conflicts occur in our lives. How much do you agree or disagree with each of the following statements?	How Much Do You Approve or Disapprove of…the National Rifle Association?					
	Do Not Approve		Somewhat Approve		Strongly/Very Strongly Approve	
	Unweighted *n*	Weighted % (95% CI)	Unweighted *n*	Weighted % (95% CI)	Unweighted *n*	Weighted % (95% CI)
Given enough provocation, I may hit another person						
Do not agree	2404	70.5 (68.5, 72.5)	1453	63.5 (60.7, 66.3)	1527	57.1 (54.5, 59.8)
Somewhat agree	787	21.8 (19.9, 23.6)	615	26.0 (23.5, 28.5)	751	26.5 (24.2, 28.9)
Strongly or very strongly agree	201	6.5 (5.3, 7.6)	178	8.9 (7.1, 10.7)	357	14.5 (12.6, 16.5)
aPD (95% CI; q-value)	Referent		1.7 (-0.6, 4.0; 0.2)		8.1 (5.1, 11.1; <0.001 )	
There are people who pushed me so far that we came to blows.						
Do not agree	3020	87.0 (85.4, 88.6)	1897	79.1 (76.5, 81.7)	2104	74.8 (72.3, 77.2)
Somewhat agree	263	7.8 (6.6, 9.0)	245	11.9 (9.9, 14.0)	329	13.7 (11.8, 15.6)
Strongly or very strongly agree	107	3.7 (2.8, 4.6)	103	7.1 (5.3, 8.9)	203	9.8 (8.1, 11.6)
aPD (95% CI; q-value)	Referent		3.6 (1.2, 6.0; 0.005)		6.8 (4.0, 9.5; <0.001 )	
I have threatened people I know.						
Do not agree	3210	92.4 (91.1, 93.6)	2064	86.2 (83.8, 88.6)	2401	85.7 (83.6, 87.9)
Somewhat agree	123	4.1 (3.2, 5.1)	137	8.3 (6.4, 10.2)	157	7.4 (5.9, 8.9)
Strongly or very strongly agree	58	2.2 (1.5, 2.9)	46	3.9 (2.4, 5.5)	83	5.4 (3.8, 6.9)
aPD (95% CI; q-value)	Referent		2.1 (0.1, 4.1; 0.07)		4.0 (1.8, 6.2; 0.001)	
At times I feel I have gotten a raw deal out of life.						
Do not agree	2416	65.9 (63.7, 68.0)	1585	65.2 (62.4, 68.0)	1817	62.5 (59.8, 65.1)
Somewhat agree	753	24.8 (22.8, 26.8)	525	24.7 (22.2, 27.2)	558	22.9 (20.6, 25.1)
Strongly or very strongly agree	215	7.5 (6.3, 8.7)	138	8.5 (6.7, 10.4)	260	12.7 (10.8, 14.6)
aPD (95% CI; q-value)			0.6 (-1.9, 3.0; 0.65)		5.0 (1.8, 8.3; 0.004)	
Other people always seem to get the breaks.						
Do not agree	2521	68.0 (65.9, 70.2)	1565	61.4 (58.5, 64.3)	1727	56.4 (53.7, 59.1)
Somewhat agree	700	24.4 (22.4, 26.4)	568	29.7 (27.0, 32.4)	678	29.5 (27.0, 32.0)
Strongly or very strongly agree	167	6.2 (5.1, 7.3)	111	7.3 (5.6, 9.0)	228	12.3 (10.3, 14.3)
aPD (95% CI; q-value)	Referent		0.9 (-1.5, 3.3; 0.52)		6.9 (3.8, 10.0; <0.001 )	
I wonder why sometimes I feel so bitter about things.						
Do not agree	2422	67.6 (65.5, 69.7)	1544	63.9 (61.1, 66.8)	1840	65.0 (62.4, 67.6)
Somewhat agree	791	24.8 (22.9, 26.7)	599	27.3 (24.7, 29.9)	588	23.6 (21.3, 26.0)
Strongly or very strongly agree	175	6.2 (5.0, 7.3)	105	7.3 (5.5, 9.0)	209	9.7 (8.1, 11.4)
aPD (95% CI; q-value)	Referent		1.3 (-1.0, 3.6; 0.31)		4.4 (1.8, 7.1; 0.003)	

Adjusted prevalence differences (aPDs) are absolute percentage point (pp) differences for “strongly or very strongly agree” responses. Models are adjusted for age (continuous), race and ethnicity (7 categories: white, non-Hispanic; Black, non-Hispanic; Hispanic, any race; Asian American/Pacific Islander, non-Hispanic; American Indian/Alaska native, non-Hispanic; 2 + races, non-Hispanic; some other race, non-), gender (3 categories: male, female, other), income (7 categories: <$10,000, $10,000-$24,999, $25,000-$49,999, $50,000-$74,999, $75,000-$99,999, $100,000-$149,999, ≥$150,000), education (5 categories: no high school diploma or GED, high school graduate/GED, some college or associate’s degree, bachelor’s degree, master’s degree or higher), Census division (9 categories: New England, Middle Atlantic, South Atlantic, East North Central, East South Central, West North Central, West South Central, Mountain, Pacific), marital status (currently married, 2 categories: no, yes), homeownership (2 categories: no, yes), political ideology (conservative, 2 categories: no, yes), rurality (urban residence, 2 categories: no, yes), alcohol consumption (drinks/week, 3 categories: 0, 1–10, ≥ 11), military service (2 categories: no, yes), and history of non-traffic arrest (2 categories: no, yes)

For a given value of the false discovery rate, Q, the q-values are chosen so that, of comparisons that reject the null hypothesis (decided by q < α), the expected proportion of these comparisons that incorrectly reject the null hypothesis is at most Q. Item non-responses are not reported in the tables but are included in the prevalence calculations

NRA approvers were more likely than non-approvers to consider violence justified in non-political circumstances where such justification was common, such as for self-defense, to prevent someone from injuring or killing another person or themselves, and to prevent property damage (Table 16). They also more frequently justified violence to win an argument or in response to an insult, but not to get respect.

**Table 16:** NRA approval and non-political violence

What do you think about the use of force or violence in the following situations?	How Much Do You Approve or Disapprove of…the National Rifle Association?					
	Do Not Approve		Somewhat Approve		Strongly/Very Strongly Approve	
	Unweighted *n*	Weighted % (95% CI)	Unweighted *n*	Weighted % (95% CI)	Unweighted *n*	Weighted % (95% CI)
In self-defense						
Never justified	61	2.2 (1.5, 2.9)	17	1.0 (0.4, 1.6)	12	0.5 (0.2, 0.9)
Sometimes justified	981	29.8 (27.8, 31.8)	371	18.7 (16.3, 21.0)	201	9.8 (7.9, 11.7)
Usually or always justified	2374	68.0 (65.9, 70.1)	1880	80.3 (77.9, 82.7)	2453	89.7 (87.8, 91.6)
aPD (95% CI; q-value)	Referent		9.8 (6.3, 13.4; <0.001 )		17.9 (14.1, 21.7; <0.001 )	
To prevent someone from injuring or killing another person						
Never justified	58	2.4 (1.6, 3.2)	16	1.0 (0.2, 1.7)	15	1.1 (0.4, 1.8)
Sometimes justified	795	25.8 (23.8, 27.8)	309	15.8 (13.6, 17.9)	172	8.9 (7.2, 10.7)
Usually or always justified	2549	71.8 (69.7, 73.9)	1935	83.3 (81.0, 85.5)	2474	90.0 (88.1, 91.9)
aPD (95% CI; q-value)	Referent		9.3 (6.0, 12.6; <0.001 )		15.0 (11.4, 18.7; <0.001 )	
To prevent someone from injuring or killing themselves						
Never justified	228	7.9 (6.6, 9.2)	94	4.1 (3.0, 5.2)	132	5.4 (4.0, 6.8)
Sometimes justified	1311	39.8 (37.7, 42.0)	767	34.7 (31.9, 37.4)	704	26.4 (24.1, 28.8)
Usually or always justified	1870	52.3 (50.0, 54.5)	1404	61.2 (58.5, 64.0)	1824	68.2 (65.7, 70.7)
aPD (95% CI; q-value)	Referent		6.3 (2.3, 10.2; 0.003)		12.9 (8.6, 17.1; <0.001 )	
To prevent harm or damage to property						
Never justified	765	23.5 (21.6, 25.4)	242	11.5 (9.7, 13.4)	201	8.4 (6.9, 9.9)
Sometimes justified	1860	54.3 (52.0, 56.5)	1197	52.2 (49.3, 55.0)	1092	41.5 (38.9, 44.1)
Usually or always justified	782	22.2 (20.3, 24.1)	824	36.3 (33.6, 39.0)	1370	50.1 (47.5, 52.8)
aPD (95% CI; q-value)	Referent		10.0 (6.4, 13.7; <0.001 )		22.6 (18.4, 26.8; <0.001 )	
To win an argument						
Never justified	3176	91.4 (90.1, 92.8)	2045	87.7 (85.6, 89.7)	2362	85.5 (83.3, 87.6)
Sometimes justified	174	6.0 (4.8, 7.1)	172	8.9 (7.2, 10.7)	227	10.1 (8.3, 11.9)
Usually or always justified	62	2.6 (1.7, 3.4)	48	3.4 (2.1, 4.7)	74	4.4 (3.1, 5.8)
aPD (95% CI; q-value)	Referent		1.7 (-0.2, 3.6; 0.09)		2.5 (0.4, 4.6; 0.03)	
In response to an insult						
Never justified	3074	87.0 (85.3, 88.6)	1965	83.0 (80.7, 85.4)	2301	81.9 (79.6, 84.2)
Sometimes justified	280	10.5 (9.0, 12.0)	232	12.5 (10.5, 14.4)	257	11.9 (10.0, 13.9)
Usually or always justified	60	2.5 (1.7, 3.3)	68	4.5 (3.1, 6.0)	103	6.2 (4.6, 7.7)
aPD (95% CI; q-value)	Referent		2.8 (0.8, 4.8; 0.009)		4.5 (2.2, 6.8; <0.001 )	
To get respect						
Never justified	3191	91.6 (90.3, 93.0)	2077	88.7 (86.6, 90.9)	2414	86.8 (84.7, 88.9)
Sometimes justified	148	5.2 (4.2, 6.3)	131	7.3 (5.6, 9.1)	167	7.7 (6.2, 9.3)
Usually or always justified	75	3.1 (2.2, 4.0)	58	3.9 (2.6, 5.2)	85	5.4 (3.9, 6.9)
aPD (95% CI; q-value)	Referent		1.0 (-1, 3; 0.34)		2.0 (-0.5, 4.4; 0.12)	

Adjusted prevalence differences (aPDs) are absolute percentage point (pp) differences for “usually or always justified” responses. Models are adjusted for age (continuous), race and ethnicity (7 categories: white, non-Hispanic; Black, non-Hispanic; Hispanic, any race; Asian American/Pacific Islander, non-Hispanic; American Indian/Alaska native, non-Hispanic; 2 + races, non-Hispanic; some other race, non-), gender (3 categories: male, female, other), income (7 categories: <$10,000, $10,000-$24,999, $25,000-$49,999, $50,000-$74,999, $75,000-$99,999, $100,000-$149,999, ≥$150,000), education (5 categories: no high school diploma or GED, high school graduate/GED, some college or associate’s degree, bachelor’s degree, master’s degree or higher), Census division (9 categories: New England, Middle Atlantic, South Atlantic, East North Central, East South Central, West North Central, West South Central, Mountain, Pacific), marital status (currently married, 2 categories: no, yes), homeownership (2 categories: no, yes), political ideology (conservative, 2 categories: no, yes), rurality (urban residence, 2 categories: no, yes), alcohol consumption (drinks/week, 3 categories: 0, 1–10, ≥ 11), military service (2 categories: no, yes), and history of non-traffic arrest (2 categories: no, yes)

For a given value of the false discovery rate, Q, the q-values are chosen so that, of comparisons that reject the null hypothesis (decided by q < α), the expected proportion of these comparisons that incorrectly reject the null hypothesis is at most Q. Item non-responses are not reported in the tables but are included in the prevalence calculations

NRA approvers were more likely to view intimate partner violence as justified in response to a wide array of actions by that partner (Table 17). The sole exception was when “a partner forces you to have sex with him or her,” in which case NRA approvers were non-significantly less likely than non-approvers to view violence as justified (approvers 28.4%, 95% CI 26.0%, 30.8%; non-approvers 31.6%, 95% CI 29.5%, 33.7%; aPD 0.3pp, 95% CI -3.6pp, 4.2pp; q = 0.92).

**Table 17:** NRA approval and justification for intimate partner violence

How much do you agree or disagree with the following reasons why it might be understandable that someone could hit a partner?	How Much Do You Approve or Disapprove of…the National Rifle Association?						
	Do Not Approve		Somewhat Approve		Strongly/Very Strongly Approve		
	Unweighted *n*	Weighted % (95% CI)	Unweighted *n*	Weighted % (95% CI)	Unweighted *n*	Weighted % (95% CI)	
A partner hurts your child, either physically or emotionally.							
Do not agree	1676	48.5 (46.3, 50.8)	1088	45.0 (42.2, 47.7)	1272	46.2 (43.5, 48.8)	
Somewhat agree	974	27.6 (25.6, 29.6)	614	27.9 (25.3, 30.5)	611	22.8 (20.6, 25.0)	
Strongly or very strongly agree	730	22.4 (20.5, 24.3)	532	25.3 (22.9, 27.8)	738	29.0 (26.5, 31.4)	
aPD (95% CI; q-value)	Referent		4.7 (1.3, 8.1; 0.01)		7.0 (3.1, 10.9; <0.001 )		
A partner hits you first.							
Do not agree	1931	55.8 (53.6, 58.0)	1243	52.4 (49.6, 55.2)	1519	54.2 (51.6, 56.9)	
Somewhat agree	952	27.1 (25.2, 29.1)	651	27.5 (25.0, 30.0)	602	23.0 (20.7, 25.2)	
Strongly or very strongly agree	487	15.2 (13.6, 16.9)	337	17.9 (15.6, 20.2)	503	21.0 (18.8, 23.3)	
aPD (95% CI; q-value)	Referent		4.0 (0.9, 7.1; 0.02)		6.3 (2.9, 9.7; <0.001 )		
A partner cheats on you.							
Do not agree	3013	86.2 (84.6, 87.8)	1929	80.8 (78.3, 83.3)	2214	78.5 (76.1, 80.8)	
Somewhat agree	260	8.7 (7.3, 10.0)	206	11.0 (9.0, 13.0)	250	11.3 (9.4, 13.2)	
Strongly or very strongly agree	111	3.7 (2.8, 4.6)	96	6.2 (4.6, 7.8)	163	8.5 (6.8, 10.2)	
aPD (95% CI; q-value)	Referent		3.1 (1.0, 5.2; 0.007)		5.9 (3.1, 8.7; <0.001 )		
A partner steals from you.							
Do not agree	3060	88.5 (87.0, 89.9)	1972	84.1 (81.8, 86.4)	2250	81.7 (79.4, 83.9)	
Somewhat agree	227	6.5 (5.4, 7.6)	187	9.4 (7.6, 11.3)	228	9.4 (7.7, 11.1)	
Strongly or very strongly agree	92	3.5 (2.6, 4.4)	71	4.5 (3.1, 5.8)	145	7.1 (5.4, 8.7)	
aPD (95% CI; q-value)	Referent		2.3 (0.2, 4.4; 0.04)		5.4 (2.6, 8.2; <0.001 )		
A partner is drunk or using drugs.							
Do not agree	2922	84.4 (82.7, 86)	1837	79.3 (76.9, 81.7)	2130	77.6 (75.3, 80.0)	
Somewhat agree	324	10.0 (8.6, 11.4)	295	12.9 (11.0, 14.9)	333	12.7 (10.9, 14.4)	
Strongly or very strongly agree	134	4.1 (3.2, 5.0)	97	5.9 (4.4, 7.3)	169	8.2 (6.4, 10.0)	
aPD (95% CI; q-value)	Referent		2.7 (0.7, 4.7; 0.01)		4.9 (2.3, 7.6; <0.001 )		
A partner embarrassed or belittled you in front of others.							
Do not agree	3117	90.1 (88.8, 91.5)	2035	86.7 (84.5, 88.9)	2302	82.4 (80.1, 84.7)	
Somewhat agree	196	6.0 (4.9, 7.0)	145	8.3 (6.5, 10.1)	219	10.1 (8.2, 11.9)	
Strongly or very strongly agree	68	2.4 (1.7, 3.2)	63	3.5 (2.3, 4.6)	113	5.9 (4.4, 7.3)	
aPD (95% CI; q-value)	Referent		2.3 (0.6, 3.9; 0.01)		5.0 (2.6, 7.4; <0.001 )		
A partner continually nags you.							
Do not agree	3144	91.0 (89.7, 92.3)	2048	88.3 (86.3, 90.4)	2363	85.8 (83.7, 87.8)	
Somewhat agree	181	5.7 (4.6, 6.8)	135	6.5 (5.0, 8.0)	173	7.2 (5.8, 8.6)	
Strongly or very strongly agree	52	1.7 (1.2, 2.3)	50	3.4 (2.1, 4.7)	96	5.4 (3.9, 7.0)	
aPD (95% CI; q-value)	Referent		2.2 (0.7, 3.7; 0.007)		4.9 (2.6, 7.1; <0.001 )		
A partner threatened to hit you.							
Do not agree	2801	80.8 (79.0, 82.6)	1828	78.0 (75.4, 80.5)	2103	75.7 (73.3, 78.2)	
Somewhat agree	428	13.4 (11.8, 15.0)	298	14.4 (12.3, 16.6)	343	13.9 (11.8, 15.9)	
Strongly or very strongly agree	150	4.3 (3.5, 5.2)	107	5.6 (4.2, 7.1)	180	8.5 (6.9, 10.2)	
aPD (95% CI; q-value)	Referent		2.9 (0.9, 4.9; 0.007)		6.0 (3.4, 8.7; <0.001 )		
A partner tried to keep you from doing something.							
Do not agree	3004	86.9 (85.3, 88.4)	1954	83.2 (80.9, 85.5)	2246	81.7 (79.5, 83.8)	
Somewhat agree	290	8.6 (7.3, 9.9)	224	10.9 (9.0, 12.8)	259	9.9 (8.2, 11.5)	
Strongly or very strongly agree	80	2.7 (2.0, 3.5)	57	4.0 (2.6, 5.4)	124	6.7 (5.1, 8.3)	
aPD (95% CI; q-value)	Referent		1.9 (0.1, 3.6; 0.04)		5.3 (3.1, 7.6; <0.001 )		
A partner forces you to have sex with him or her.							
Do not agree	1669	45.9 (43.7, 48.2)	1163	48.0 (45.2, 50.8)	1507	52.4 (49.7, 55.0)	
Somewhat agree	704	20.8 (18.9, 22.6)	471	20.3 (18.0, 22.6)	436	17.3 (15.3, 19.3)	
Strongly or very strongly agree	997	31.6 (29.5, 33.7)	589	29.3 (26.8, 31.9)	679	28.4 (26.0, 30.8)	
aPD (95% CI; q-value)	Referent		-0.1 (-3.6, 3.5; 0.97)		0.3 (-3.6, 4.2; 0.92)		

Adjusted prevalence differences (aPDs) are absolute percentage point (pp) differences for “strongly or very strongly agree” responses. Models are adjusted for age (continuous), race and ethnicity (7 categories: white, non-Hispanic; Black, non-Hispanic; Hispanic, any race; Asian American/Pacific Islander, non-Hispanic; American Indian/Alaska native, non-Hispanic; 2 + races, non-Hispanic; some other race, non-), gender (3 categories: male, female, other), income (7 categories: <$10,000, $10,000-$24,999, $25,000-$49,999, $50,000-$74,999, $75,000-$99,999, $100,000-$149,999, ≥$150,000), education (5 categories: no high school diploma or GED, high school graduate/GED, some college or associate’s degree, bachelor’s degree, master’s degree or higher), Census division (9 categories: New England, Middle Atlantic, South Atlantic, East North Central, East South Central, West North Central, West South Central, Mountain, Pacific), marital status (currently married, 2 categories: no, yes), homeownership (2 categories: no, yes), political ideology (conservative, 2 categories: no, yes), rurality (urban residence, 2 categories: no, yes), alcohol consumption (drinks/week, 3 categories: 0, 1–10, ≥ 11), military service (2 categories: no, yes), and history of non-traffic arrest (2 categories: no, yes)

For a given value of the false discovery rate, Q, the q-values are chosen so that, of comparisons that reject the null hypothesis (decided by q < α), the expected proportion of these comparisons that incorrectly reject the null hypothesis is at most Q. Item non-responses are not reported in the tables but are included in the prevalence calculations

## Discussion

In this large, nationally representative survey of adults in the United States, more than one-fourth of respondents (27.0%) strongly or very strongly approved of the National Rifle Association in 2022. While the NRA is commonly thought of as an organization of firearm owners—and ownership may be the rule among its members—its sphere of influence is broader. Half the NRA approvers among our respondents were not personal firearm owners, and others have reported similar findings [6]. This might help explain what in the past—before a failed bankruptcy filing and a court finding of corruption among its leadership [28, 29]—was seen as the organization’s disproportionate political and social influence.

The findings indicate that it would also be incorrect to view NRA approval as a male, white, right-wing phenomenon. Nearly half the approvers identified as female, and more than 20% identified as other than white, non-Hispanic. NRA approvers among our respondents included Democrats and liberals, albeit in small numbers, and NRA approvers were more likely than non-approvers to approve of Redneck Revolt, a left-wing pro-firearms organization [45].

We found approval of the NRA to be strongly and pervasively associated with the belief that political violence is justified and with willingness to engage in political violence. This association has many potential points of origin; NRA approval was associated with a wide array of attributes and beliefs that have previously been linked to political violence in our survey cohort [19–24] and in research by others [7, 9–11, 17, 18]. At least some of these, including racism [9, 16, 17, 24], xenophobia [18, 24], and misogyny [9, 24], are resistant to change.

The findings do not suggest, and we do not imply, that involvement with the NRA creates a predisposition toward political violence that would not exist otherwise. It might well intensify and further the development of such a predisposition, however, through the creation of a shared social identity [4–7, 9, 11, 46] among individuals with attributes that are associated with an increase in support for and willingness to engage in political violence [25–27, 47].

What are the practical implications of these findings? It is important to recognize the continued potential importance of the NRA as a contributor to risk for political violence in the US. Among social movement organizations, at least, it may remain uniquely capable of mobilizing large numbers of people who are armed and willing to use violence to advance political objectives. The organization might even invoke the United States Supreme Court, which in its *Heller* decision noted with approval that “when the able-bodied men of a nation are trained in arms and organized, they are better able to resist tyranny” [48].

The NRA’s potential for mobilizing its supporters likely depends to a large degree on the extent to which it is still perceived by its members and others, principally firearm owners, to be an honest representative of their interests. That perception may have been diminished by its bankruptcy and corruption scandal [28, 29] and by a report in late 2024 from members of the organization’s leadership that “President Trump and his most inner circle have lost faith in the N.R.A” [49]. Unfortunately, an interest in regaining the president’s favor might increase the NRA’s willingness to mobilize its adherents in support of political violence, should the president request it, and the president’s own support for violence against civilians is well established [50–52].

Here it is important to note that most NRA approvers among our respondents rejected political violence. Those NRA approvers—perhaps particularly the firearm owners among them—might be uniquely credible messengers in efforts to create a population-wide culture of non-acceptance for political violence. In this cohort [53] and as suggested by other research [54], suasion by trusted family members, friends, and other community members may prevent political violence—just as it produces positive changes in other health behaviors.

### Limitations

The findings are subject to sampling error and bias due to nonresponse, social desirability, and other factors. NRA approval status was collected in 2022, after the organization’s failed attempt to file for bankruptcy in 2021 [28] but before court verdicts in New York in 2024 found the organization and its senior leadership guilty of financial misconduct [29]. NRA approval is likely less prevalent now than in 2022. That attrition would have been selective, and findings on the measures included in this study might well have changed. We did not collect data on NRA membership and expect that our respondents include non-members who approve of the organization and, given the organization’s recent history, members who do not. Given the breadth of measures included here, our findings are susceptible to the effects of a broad array of external events: mass shootings, war in Ukraine and elsewhere, conflict in the US centering on race and ethnicity and immigration status, and others. Where we have used standardized scales, choosing different scales might have produced different findings. Similarly, estimates of support for violence to advance political objectives may be sensitive to the specific objectives presented.

## Conclusion

Findings from this large, nationally representative survey suggest that approval of the NRA, a large social movement organization, is associated with and increase in support for political violence and willingness to engage in it. These findings and the NRA’s known ability to generate political activity among its supporters should be considered in formulating plans to prevent political violence.

## Electronic Supplementary Material

Supplementary Material 1

## Data Availability

The datasets generated and/or analyzed during the current study are not publicly available as analyses are continuing but will be made available to qualified researchers subject to the terms of a data use agreement.

## Abbreviations

aPD: Adjusted prevalence difference
CI: Confidence interval
PP: percentage point
SD: Standard deviation
