# Supplementary material for: Approval of the National Rifle Association and political violence: findings from a nationally representative survey
Source: Inj Epidemiol. 2026 May 14;13:40. doi: 10.1186/s40621-026-00685-2 (PMC13196074; doi:10.1186/s40621-026-00685-2)
Supplement: Supplementary file 1 — Supplementary Material 1 [file 40621_2026_685_MOESM1_ESM.pdf]

Supplement

Approval of the National Rifle Association and Political Violence:

Findings from a Nationally Representative Survey

Garen J. Wintemute, MD, MPH; Yueju Li, MA; Aaron B. Shev, PhD; Sonia L. Robinson, PhD, MPH; Elizabeth A. Tomsich, PhD; Mona A. Wright, MPH; Veronica A. Pear, PhD, MPH, MA

This supplement has been provided by the authors to give readers additional information about the work.

Page	Title
2	Questions that supplied data for this study
17	Additional methods text
22	Additional results text
23	References
25	Table S1. Sociodemographic characteristics of respondents
27	Table S2. Sociodemographic characteristics (unweighted) of respondents and non-respondents in the 2022 and 2023 surveys

QUESTIONS THAT SUPPLIED DATA FOR THIS STUDY

Response options are presented here in order from negative to positive (e.g., “not important” to “extremely important”). Respondents were randomized 1:1 to receive responses in that order or the reverse.
Data were collected in 2023 unless specified otherwise. Questions or items that were repeated or adapted from prior surveys by other investigators contain citations to those surveys.

Democracy, authoritarianism, and elections

Now we’d like to ask you a few questions about the United States as you see it now, in 2023.

Q: When thinking about democracy in the United States these days, do you believe…?[1]

1. There is a serious threat to our democracy.
2. There may be a threat to our democracy, but it is not serious.
3. There is no threat to our democracy.

Q: Please indicate whether you believe each of the following is a threat to democracy in the United States. Is it a serious threat; a threat, but not a serious threat; or not a threat?

a. The influence of big money in elections[2]
b. The influence of foreign governments in elections
c. The possibility of political violence[2]
d. The influence of white nationalist groups
e. The influence of groups like Black Lives Matter
f. Efforts to overturn the results of elections

1. A serious threat
2. A threat, but not a serious threat
3. Not a threat

Q: How important do you think it is for the United States to remain a democracy?[3]

1. Not important
2. Somewhat important
3. Very important
4. Extremely important

Q: How much do you agree or disagree with the following statements about democracy in the United States?

a. Democracy is the best form of government.[4]
b. These days, American democracy only serves the interests of the wealthy and powerful.[5]

c. Having a strong leader for America is more important than having a democracy.
d. The 2020 election was stolen from Donald Trump, and Joe Biden is an illegitimate president.
e. We should suspend Congress for a few years so a strong leader can clean up the mess made by politicians in Washington.[6]

1. Do not agree
2. Somewhat agree
3. Strongly agree
4. Very strongly agree

(Asked in 2022.)
Q: People have many different views about American society. How much do you agree or disagree with each of the following?

h. Armed citizens should patrol polling places at election time.

1. Do not agree
2. Somewhat agree
3. Strongly agree
4. Very strongly agree

Q: Which is more important to you…?[2]

1a. Having election outcomes determined democratically
OR
1b. Having political leaders I can trust to look out for my values and interests

Partisan identity and political ideology

Q: Generally speaking, do you think of yourself as...Select one answer only.

1. Republican
2. Democrat
3. Independent
6. Something else

(Asked if Republican)
Q: Would you call yourself a...Select one answer only.

1. Strong Republican
2. Not very strong Republican

(Asked if Democrat)
Q: Would you call yourself a...Select one answer only.

1. Strong Democrat
2. Not very strong Democrat

(Asked if Independent or Something else)
Q: Do you think of yourself as closer to the...Select one answer only.

1. Republican Party
2. Democratic Party
3. Do not lean either way

(Asked if Republican OR Leans Republican)
Q: Do you think of yourself as a MAGA Republican?

1. No
2. Yes

(Asked if Not MAGA Republican OR Democrat / Leans Democrat)
Q: Do you think of yourself as a supporter of the MAGA movement?

1. No
2. Yes

(Asked if Republican OR Leans Republican)
Q: When you think about Democrats, do you tend to think of them as…[7]

a. Political opposition – that is, if they win, you just won't get the policies you want
OR
b. Enemies – that is, if they win, your life or your entire way of life may be threatened

(Asked if Democrat OR Leans Democrat)
Q: When you think about Republicans, do you tend to think of them as…[7]
a. Political opposition - that is, if they win, you just won't get the policies you want
OR
b. Enemies - that is, if they win, your life or your entire way of life may be threatened

Q: In general, do you think of yourself as...

1. Extremely liberal
2. Liberal
3. Slightly liberal
4. Moderate/middle of the road
5. Slightly conservative
6. Conservative
7. Extremely conservative

Hatred, fear, and enmity toward others

Homonegativity

Q: How much do you agree or disagree with each of the following statements about gay men and lesbian women in the United States today?[8]

a. Celebrations such as "Gay Pride Day" are ridiculous, because they assume that an individual's sexual orientation should constitute a source of pride.
b. Gay men and lesbian women should stop shoving their lifestyle down other people's throats.
c. Many gay men and lesbian women use their sexual orientation so that they can obtain special rights and privileges.
d. Gay men and lesbian women who are "out of the closet" should be admired for their courage.
e. In today's tough economic times, Americans' tax dollars shouldn't be used to support gay and lesbian organizations.
f. Gay men and lesbian women should stop complaining about the way they are treated in society, and simply get on with their lives.

1. Do not agree
2. Somewhat agree
3. Strongly agree
4. Very strongly agree

Racism

Q: How much do you agree or disagree with each of the following statements about people in the United States today?

a. White people benefit from advantages in society that Black people do not have.[9] (Reverse coded.)
b. Discrimination against whites is as big a problem as discrimination against Blacks and other minorities.[10]
c. A group of people in this country is trying to replace native-born Americans with immigrants and people of color who share their political views.
d. Having more Black Americans, Latinos, and Asian Americans is good for the country.[11] (Reverse coded.)

1. Do not agree
2. Somewhat agree
3. Strongly agree
4. Very strongly agree

Transphobia

Q: How much do you agree or disagree with each of the following statements about gender in the United States today?[12]

a. I think there is something wrong with a person who says that they are neither a man nor a woman.
b. I would be upset, if someone I'd known a long time revealed to me that they used to be another gender.
c. I avoid people on the street whose gender is unclear to me.
d. When I meet someone, it is important for me to be able to identify them as a man or a woman.
e. I believe that the male/female dichotomy is natural.
f. I believe that a person can never change their gender.

1. Do not agree
2. Somewhat agree
3. Strongly agree
4. Very strongly agree

Xenophobia

Q: How much do you agree or disagree with each of the following statements about people who have immigrated to the United States?[13]

a. Interacting with immigrants makes me uneasy.
b. Immigrants cause an increase in crime.
c. I enjoy interacting with immigrants.
d. I am afraid that our own culture will be lost with an increase in immigration.
e. I am afraid that in case of political tension, immigrants will be loyal to their country of origin.

1. Do not agree
2. Somewhat agree
3. Strongly agree
4. Very strongly agree

Hostile Sexism

Q: How much do you agree or disagree with each of the following statements about women in the United States today?[14]

a. Women seek to gain power by getting control over men.
b. Women exaggerate problems they have at work.
c. Once a woman gets a man to commit to her, she usually tries to put him on a tight leash.

d. When women lose to men in a fair competition, they typically complain about being discriminated against.
e. Many women get a kick out of teasing men by seeming sexually available and then refusing male advances.
f. Feminists are making unreasonable demands of men.

1. Do not agree
2. Somewhat agree
3. Strongly agree
4. Very strongly agree

Islamophobia

Q: How much do you agree or disagree with each of the following statements about people's religious beliefs in the United States today?[15]

a. Most Muslims living in the United States are more prone to violence than other people.
b. Most Muslims living in the United States discriminate against women.
c. Most Muslims living in the United States are hostile to the United States.
d. Most Muslims living in the United States are less civilized than other people.

1. Do not agree
2. Somewhat agree
3. Strongly agree
4. Very strongly agree

Antisemitism

Q: How much do you agree or disagree with each of the following statements about people's religious beliefs in the United States today?[16]

a. Jewish people can be trusted just as much as other Americans in business.
b. Jewish people are just as loyal to the United States as other Americans.
c. Compared to other groups, Jewish people have too much power in the media.
d. Jewish people talk about the Holocaust just to further their political agenda.
e. Jewish people chase money more than other people do.

1. Do not agree
2. Somewhat agree
3. Strongly agree
4. Very strongly agree

Conspiracism

Q: There is often debate about whether the public is told the whole truth about various important issues. How much do you agree or disagree with the following statements?[17]

a. The government is involved in the murder of innocent citizens and/or well-known public figures and keeps this a secret.
b. The spread of certain viruses and/or diseases is the result of the deliberate, concealed efforts of some organization.
c. The government permits or perpetrates acts of terrorism on its own soil, disguising its involvement.
d. Technology with mind-control capacities is used on people without their knowledge.
e. The government falsely blames innocent people to hide its involvement in criminal activity.
f. Experiments involving new drugs or technologies are routinely carried out on the public without their knowledge or consent.

1. Do not agree
2. Somewhat agree
3. Strongly agree
4. Very strongly agree

QAnon and Christian nationalism

Q: People have many different views about society in the United States. How much do you agree or disagree with each of the following?

a. The government, media, and financial worlds in the U.S. are controlled by a group of Satan-worshipping pedophiles who run a global child sex trafficking operation.[18]
b. There is a storm coming soon that will sweep away the elites in power and restore the rightful leaders.[18]
c. The chaos in America today is evidence that we are living in what the Bible calls "the end times."[19]

1. Do not agree
2. Somewhat agree
3. Strongly agree
4. Very strongly agree

Q: How much do you agree or disagree with each of the following statements about people's religious beliefs in the United States today?[20]

a. The U.S. government should declare America a Christian nation.
b. U.S. laws should be based on Christian values.
c. If the U.S. moves away from our Christian foundations, we will not have a country anymore.

d. Being Christian is an important part of being truly American.
e. God has called Christians to exercise dominion over all areas of American society.

1. Do not agree
2. Somewhat agree
3. Strongly agree
4. Very strongly agree

Extremist organizations and social movements (asked in 2022)

Q: How much do you approve or disapprove of these named groups and organizations?

a. Proud Boys
b. Oath Keepers
c. Three Percenters
d. QAnon
e. National Rifle Association

1. Do not approve
2. Somewhat approve
3. Strongly approve
4. Very strongly approve
5. I don't know enough about this group or organization to rate it
6. I have never heard of this group or organization

Q: How much do you approve or disapprove of these political or social movements?

a. The militia movement
b. The antifascist (Antifa) movement
c. The white supremacy movement
d. The Christian nationalist movement
e. The boogaloo movement
f. The anarchist movement

1. Do not approve
2. Somewhat approve
3. Strongly approve
4. Very strongly approve
5. I don't know enough about this political or social movement to rate it
6. I have never heard of this political or social movement

Firearm ownership and use (asked in 2022)

Q: Do you happen to keep any guns in your home or garage?

1. Yes
2. No

(Question asked if the response to the prior question was "yes.")
Q: Do any of these guns personally belong to you?

1. Yes
2. No

(Question asked if the response to the prior question was "yes." Presented with firearm types in rows and responses in columns. Respondents gave separate answers for each firearm type.)
Q: Do you personally own any of the following types of guns?

a. Handguns
b. Rifles
c. Shotguns
d. Other types of guns

    1. Yes
    2. No

(Question asked if the response to "rifles" in the prior question was "yes.")
Q: Do you own any rifles of the type sometimes called tactical rifles, or modern sporting rifles, or assault rifles, such as an AR-15, an AK-47, or an SKS?

1. Yes
2. No

(Question asked if the response to the personal ownership question was "yes.")
Q: Did you buy any guns this year, in 2022?

1. Yes
2. No

Q: Did you buy any guns in 2021?

1. Yes
2. No

Q: Did you buy any guns in 2020?

1. Yes
2. No

Q: When you bought guns in (insert earliest year from the series above) did you already own any guns?

1. Yes
2. No

(Question asked of all respondents)
Q: In the last year, have you carried a loaded gun (handgun, rifle, or shotgun) on your person when you were out in public? Do not include hunting, time at a shooting range, or similar activities.

1. Yes
2. No

(Question asked if the response to the prior question was "yes.")
Q: In the last year, and on days when you were out in public, how often have you carried a loaded gun on your person? Again, do not include hunting, time at a shooting range, or similar activities.

1. Not often at all
2. Less than half the time
3. About half the time
4. More than half the time
5. All (or nearly all) the time

Non-political aggression and violence

Trait aggression

Q: Sometimes disagreements and conflicts occur in our lives. The next items are about ways you might respond to some of those things. How much do you agree or disagree with each of the following statements?[21]

a. Given enough provocation, I may hit another person.
b. There are people who pushed me so far that we came to blows.
c. I have threatened people I know.
d. At times I feel I have gotten a raw deal out of life.
e. Other people always seem to get the breaks.
f. I wonder why sometimes I feel so bitter about things.

1. Do not agree
2. Somewhat agree
3. Strongly agree
4. Very strongly agree

Violence in specific nonpolitical circumstances (asked in 2022)

Now we have a few questions about the use of force or violence. "Force or violence" means physical force strong enough that it could cause pain or injury to a person. A reminder: your responses will be kept confidential and anonymous.

Q: In general, what do you think about the use of force or violence in the following situations—is it never justified, sometimes justified, usually justified, or always justified? "Force or violence" means physical force strong enough that it could cause pain or injury to a person.

a. In self defense
b. To prevent someone from injuring or killing another person
c. To prevent someone from injuring or killing themselves
d. To prevent harm or damage to property
e. To win an argument
f. In response to an insult
g. To get respect

1. Never justified
2. Sometimes justified
3. Usually justified
4. Always justified

Intimate partner violence

Most people agree that getting physical (hitting, slapping, etc.) with a partner is not a good idea, but sometimes this happens. How much do you agree or disagree with the following reasons why it might be understandable that someone could hit a partner?[22]

a. A partner hurts your child, either physically or emotionally.
b. A partner hits you first.
c. A partner cheats on you.
d. A partner steals from you.
e. A partner is drunk or using drugs.
f. A partner embarrassed or belittled you in front of others.
g. A partner continually nags you.
h. A partner threatened to hit you.
i. A partner tried to keep you from doing something.
j. A partner forces you to have sex with him or her.

1. Do not agree
2. Somewhat agree
3. Strongly agree
4. Very strongly agree

Political violence

Q: People have different views about violence in the United States. How much do you agree or disagree with each of the following statements?

a. If elected leaders will not protect American democracy, the people must do it themselves, even if it requires taking violent actions.[5]
b. Because things have gotten so far off track, true American patriots may have to resort to violence in order to save our country.[18]
c. Our American way of life is disappearing so fast that we may have to use force to save it.[5]

1. Do not agree
2. Somewhat agree
3. Strongly agree
4. Very strongly agree

Now we have a few questions about the use of force or violence. "Force or violence" means physical force strong enough that it could cause pain or injury to a person. A reminder: your responses will be kept confidential and anonymous.

Q: People sometimes talk about using force or violence to achieve political objectives. In general, what do you think about using force or violence to advance an important political objective that you support—is it…?

1. Never justified
2. Sometimes justified
3. Usually justified
4. Always justified

Q: Your view of the use of force or violence to advance an important political objective might depend on the specific objective that was involved. What do you think about the use of force or violence in the following situations—is it never justified, sometimes justified, usually justified, or always justified?

a. To return Donald Trump to the presidency this year
b. To stop an election from being stolen
c. To stop people who do not share my beliefs from voting
d. To prevent discrimination based on race or ethnicity

e. To preserve an American way of life based on Western European traditions
f. To oppose the government when it does not share my beliefs
g. To oppose the government when it tries to take private land for public purposes
h. To protect the environment or stop climate change
i. To protect the rights of animals

1. Never justified
2. Sometimes justified
3. Usually justified
4. Always justified

Q: You said that in general, the use of force or violence was [response inserted] to advance an important political objective that you support. Your opinion might depend on the specific objective that was involved. What do you think about the use of force or violence in the following situations—is it never justified, sometimes justified, usually justified, or always justified?

a. To stop voter fraud
b. To stop voter intimidation
c. To stop police violence
d. To reinforce the police
e. To stop illegal immigration
f. To keep our borders open
g. To stop a protest or demonstration
h. To support a protest or demonstration
i. To preserve the American way of life I believe in
j. To oppose Americans who do not share my beliefs

1. Never justified
2. Sometimes justified
3. Usually justified
4. Always justified

(Questions asked of respondents who endorsed at least 1 use of violence to achieve a specific political objective.)

The next questions are about your personal willingness to use force or violence.
Q: You agreed that the use of force or violence could be justified to advance [one/some] of the political objectives we just discussed. In [that situation/those situations], how willing would you personally be to…

a. Use force or violence as part of a group of people who share your beliefs
b. Use force or violence on your own, as an individual
c. Organize a group of people who share your beliefs to use force or violence

1. Not willing
2. Somewhat willing
3. Very willing
4. Completely willing

Q: In a situation where you think force or violence is justified to advance an important political objective, how willing would you personally be to use force or violence in each of these ways?

a. To damage property
b. To threaten or intimidate a person
c. To injure a person
d. To kill a person

1. Not willing
2. Somewhat willing
3. Very willing
4. Completely willing

Q: In a situation where you think force or violence is justified to advance an important political objective, how willing would you personally be to use force or violence against a person because they are…

a. An elected federal or state government official
b. An elected local government official
c. A public health official
d. A member of the military or National Guard
e. A police officer
f. A person who does not share your race or ethnicity
g. A person who does not share your religion
h. An election worker, such as a poll worker or vote counter
i. A person who does not share your political beliefs
j. A member of the Democratic Party
k. A member of the Republican Party

1. Not willing
2. Somewhat willing
3. Very willing
4. Completely willing

(Questions asked of all respondents.)
Q: Thinking now about the future and all the changes it might bring, how likely is it that you will use a gun in any of the following ways in the next few years—in a situation where you think force or violence is justified to advance an important political objective?

a. I will be armed with a gun.
b. I will carry a gun openly, so that people know I am armed.
c. I will threaten someone with a gun.
d. I will shoot someone with a gun.

1. Not likely
2. Somewhat likely
3. Very likely
4. Extremely likely

Q: Some people talk about a second civil war in the United States. Which of the following comes closer to your view of what a second civil war might look like?

a. A second civil war would be like the first Civil War in the United States, with opposing armies and large battles.
OR
b. A second civil war would be like an insurgency or guerrilla war, with small groups attacking specific targets or people.

Q: How much do you agree or disagree with each of the following statements?

a. In the next few years, there will be civil war in the United States.
b. The United States needs a civil war to set things right.

1. Do not agree
2. Somewhat agree
3. Strongly agree
4. Very strongly agree

ADDITIONAL METHODS TEXT

Measures

Survey items that supplied data for the 7 forms of hatred, fear, and enmity toward others we included were obtained in all but 1 case from validated scales.[8,12-16] These scales were selected based on recency of development, extent of validation, and applicability to estimating associations between the phobias they measured and political violence. Questionnaire length limitations precluded use of complete scales; we selected items for inclusion based on their direct relevance to violence. An abbreviated validated scale for racism[23] was included in the questionnaire, but the selected items did not have an acceptable Cronbach's α (a measure of internal consistency among items in a scale).[24] Analyses for racism relied instead on 4 items regarding racism that had a Cronbach's α of 0.81 in our 2022 survey[25] and were included in 2023.

Questions on trait aggression and intimate partner violence were also obtained from validated scales.[21,22]

Firearm ownership and use

Respondents were categorized as personal firearm owners, nonowners with firearms at home, and nonowners without firearms at home. Firearm owners were categorized in 4 groups based on the type(s) of firearm they owned: assault-type rifle owners (owns 1 or more assault-type rifles; may own firearms of other types), other rifle owners (owns 1 or more rifles that are not-assault type rifles; does not own assault-type rifles; may own firearms of other types), handgun-only owners (owns 1 or more handguns and no firearms of other types), other owners (owns any combination of handguns, shotguns, and firearms of other types; does not own

rifles). Firearm owners were also categorized in 2 groups by recency of most recent purchase: 2020 or later, and 2019 or earlier. Finally, owners were categorized in 4 groups based on their frequency of carrying a loaded firearm on their person when out in public (with sporting use excluded): never/not often at all; less than half the time/about half the time/more than half the time; and always/nearly always.

Violence was represented in the questionnaire by “force or violence,” defined as “physical force strong enough that it could cause pain or injury to a person.” “Force or violence to advance an important political objective that you support” was used in questions about respondents’ support for and willingness to engage in political violence.

Respondents were asked about the extent to which they considered political violence to be justified “in general” and then about justification for its use to advance 19 specified political objectives. Examples include “to return Donald Trump to the presidency this year” and “to stop police violence.”

Respondents who considered violence at least sometimes justified to advance at least 1 of these 19 objectives were asked about their personal willingness to engage in political violence: in 3 social contexts (alone, as a member of a group, as an organizer of a group), for 4 types of violence (to “damage property,” “threaten or intimidate a person,” “injure a person,” “kill a person”) and against 11 target populations (examples: “an elected federal or state government official,” “a police officer,” “a person who does not share your religion”).

All respondents were asked about the likelihood of their future use of firearms in a situation where they considered political violence justified (examples: “I will be armed with a

gun"; "I will shoot someone with a gun") and their view on whether civil war was likely and needed.

The text of all questionnaire items included in this analysis is in this Supplement.

Statistical analysis

Our approach to calculating scores for 7 forms of fear, hatred, and enmity toward others has been reported previously.[25] Approximately 4.3% of respondents were missing more than half of the items for at least 1 individual phobia, and approximately 7.1% of respondents were missing at least 1 item. These missing values were imputed using Multiple Imputation by Chained Equations (MICE). Individual item responses for the 7 phobias were then coded ordinally (e.g., do not agree = 0, somewhat agree = 1, strongly agree = 2, very strongly agree = 3) and summed for each respondent for each phobia. Summed scores were normalized to a range from 0 to 1, with 0 and 1 representing the minimum and maximum theoretically possible scores. Normalized scores were then categorized according to their position on that range (e.g., strong agreement, normalized score > 0.66… and ≤ 1; moderate agreement, normalized score > 0.33… and ≤ 0.66; weak agreement, normalized score > 0 and ≤ 0.33…; non-agreement, normalized score = 0).

The adjusted model used for computing prevalence differences was developed for a prior analysis of the association between firearm ownership and political violence in this cohort.[26] In that analysis, variables were selected for inclusion based on concordance with theory, findings from prior research, and fit statistics.

Variables entered into the models were coded as follows: age (continuous), race and ethnicity (7 categories: white, non-Hispanic; Black, non-Hispanic; Hispanic, any race; Asian American/Pacific Islander, non-Hispanic; American Indian/Alaska native, non-Hispanic; 2+ races, non-Hispanic; some other race, non-), gender (3 categories: male, female, other), income (7 categories: <$10,000, $10,000-$24,999, $25,000-$49,999, $50,000-$74,999, $75,000-$99,999, $100,000-$149,999, ≥$150,000), education (5 categories: no high school diploma or GED, high school graduate/GED, some college or associate's degree, bachelor's degree, master’s degree or higher), Census division (9 categories: New England, Middle Atlantic, South Atlantic, East North Central, East South Central, West North Central, West South Central, Mountain, Pacific), marital status (currently married, 2 categories: no, yes), homeownership (2 categories: no, yes), political ideology (conservative, 2 categories: no, yes), rurality (urban residence, 2 categories: no, yes), alcohol consumption (drinks/week, 3 categories: 0, 1-10, ≥11), military service (2 categories: no, yes), and history of non-traffic arrest (2 categories: no, yes).

We examined the following models:

Model 0: unadjusted;

Model 1: adjusted for age, race, and sex;

Model 2: additionally adjusted for income, education, and census division;

Model 3: additionally adjusted for marital status, homeownership, political ideology, and rurality;

Model 4: additionally adjusted for alcohol consumption, military service, and history of non-traffic arrest;

Model 4 was selected; findings from Model 4 appear in the ‘Adjusted prevalence difference” rows in tables.

Alternative approaches to modeling exist. For example, multinomial logistic regression allows for categorical outcomes, which would allow us to model, for example, associations with “never justified”, “sometimes justified”, and “usually/always justified.” We have a different interest in this study and are using linear regression to determine the prevalence differences between “usually/always justified” and “never/sometimes justified” (or “strongly/very strongly agree” and “do not agree,” or “very/completely willing” and “not willing”) groups.

In each of those pairings, the first is plausibly the group at highest risk for violence. We are less interested in examining associations with “sometimes” or “somewhat” responses, in part because their interpretation is murky.

ADDITIONAL RESULTS TEXT

Nonresponse

One item included in this analysis had a nonresponse percentage above 2.0%:

Q: Which is more important to you…?

1a. Having election outcomes determined democratically
OR
1b. Having political leaders I can trust to look out for my values and interests

Nonresponse = 2.4%

REFERENCES

1. NPR/PBS NewsHour/Marist National Poll. Trust in elections, threat to democracy, November 2021. 2021 November 1. https://maristpoll.marist.edu/polls/npr-pbs-newshour-marist-national-poll-trust-in-elections-threat-to-democracy-biden-approval-november-2021/.
2. Hart Research Associates. The New Republic Democracy Survey (Study #14230). 2022 March. https://newrepublic.com/article/166027/democracy-poll.
3. Grinnell College National Poll. 52% of Americans believe democracy facing "major threat." Study #2243. 2021 October 20. https://www.grinnell.edu/news/52-americans-believe-democracy-facing-major-threat.
4. The Economist/YouGov Poll. 2021 June 13-16. https://docs.cdn.yougov.com/uagnfc262c/econTabReport.pdf.
5. Survey Center on American Life. January 2021 American Perspectives Survey topline questionnaire. https://www.americansurveycenter.org/wp-content/uploads/2021/03/January-2021-APS-Topline-Questionnaire.pdf.
6. Democracy Fund Voter Study Group. Guide to views of The Electorate Research Survey. 2021 December. https://www.voterstudygroup.org/data/voter-survey.
7. De Pinto J, Backus F, Salvanto A. CBS News Battleground Tracker: What do voters in each party want in a candidate? 2022 Aug 2. https://www.cbsnews.com/news/what-voters-want-in-candidate-opinion-poll-2022-08-02/.
8. Morrison MA, Morrison TG. 2011 Sexual orientation bias toward gay men and lesbian women: modern homonegative attitudes and their association with discriminatory behavioral intentions. J Appl Soc Psychol. 2011;41(11):2573-2599.
9. Pew Research Center. Deep divisions in Americans' views of nation's racial history – and how to address it. 2021 August. https://www.pewresearch.org/politics/2021/08/12/deep-divisions-in-americans-views-of-nations-racial-history-and-how-to-address-it/.
10. Cox D, Lienesch R, Jones RP. Beyond economics: fears of cultural displacement pushed the white working class to Trump | PRRI/The Atlantic Report. Public Religion Research Institute. 2019 May 17. https://www.prri.org/research/white-working-class-attitudes-economy-trade-immigration-election-donald-trump/.
11. Pew Research Center. Americans see advantages and challenges in country's growing racial and ethnic diversity. 2019 May. https://www.pewresearch.org/social-trends/2019/05/08/americans-see-advantages-and-challenges-in-countrys-growing-racial-and-ethnic-diversity/.
12. Nagoshi JL, Adams KA, Terrell HK, Hill ED, Brzuzy S, Nagoshi CT. Gender differences in correlates of homophobia and transphobia. Sex Roles. 2008;59:521-531.
13. Maloni MJ, Gligor DM, Blumentritt T, Gligor N. Fear or competition? Antecedents to U.S. business student immigration attitudes. J Manag Educ. 2022;46(4):715-750.
14. Glick P, Fiske ST. Hostile and benevolent sexism: measuring ambivalent sexist attitudes toward women. Psychol Women Q. 1997;21:119-135.
15. Institute for Social Policy and Understanding. The National American Islamophobia Index. Undated. https://www.ispu.org/islamophobia-index/.
16. Allington D, Hirsch D, Katz L. The generalised antisemitism (geas) scale: validity and factor structure. J Contemp Antisemitism. 2022;5(2):1-28.

17. Brotherton R, French CC, Pickering AD. Measuring belief in conspiracy theories: the generic conspiracist beliefs scale. Front Psychol. 2013;4:279.
18. Public Religion Research Institute. The persistence of Q-Anon in the post-Trump era: an analysis of who believes the conspiracies. 2022 Feb 24. https://www.prri.org/research/the-persistence-of-qanon-in-the-post-trump-era-an-analysis-of-who-believes-the-conspiracies/.
19. IFYC – PRRI Survey on Religion & COVID-19 Vaccine Trust. 2021 March. https://www.prri.org/wp-content/uploads/2021/05/Topline-IFYC-PRRI-Survey-on-Religion-and-COVID-19-Vaccine-Trust-v2_final.pdf.
20. Public Religion Research Institute and The Brookings Institution. A Christian nation? Understanding the threat of Christian nationalism to American democracy and culture. 2023 Feb 8. https://www.prri.org/research/a-christian-nation-understanding-the-threat-of-christian-nationalism-to-american-democracy-and-culture/.
21. Diamond PM, Magaletta PR. The Short-Form Buss-Perry Aggression Questionnaire (BPAQ-SF): a validation study with federal offenders. Assessment. 2006;13(3):227-240.
22. Copp JE, Giordano PC, Longmore MA, Manning WD. The development of attitudes toward intimate partner violence: an examination of key correlates among a sample of young adults. J Interpers Violence. 2019;34(7):1357-1387.
23. Uhlmann EL, Brescoll VL, Machery E. The motives underlying stereotype-based discrimination against members of stigmatized groups. Soc Justice Res 2010;23(1): 1-16.
24. Cronbach LJ. Coefficient alpha and the internal structure of tests. Psychometrika 1951;16(3):297-334.
25. Wintemute GJ, Velasquez B, Tomsich EA, Reeping PM, Robinson SL, Tancredi D, Pear VA. Fear, loathing, and political violence in the United States: findings from a nationally representative survey. Lancet Reg Health Am. 2025;51:101235.
26. Wintemute GJ, Crawford A, Robinson SL, Tomsich EA, Reeping PM, Schleimer JP, Pear VA. Firearm ownership and support for political violence in the United States. JAMA Netw Open. 2024;7(4):e243623.

Table S1. Sociodemographic characteristics of respondents

Characteristic	All Wave 2 Respondents		Included in the Analytic Sample (n = 8361): How Much Do You Approve or Disapprove of…the National Rifle Association? Do Not Approve		Somewhat Approve		Strongly/Very Strongly Approve		Excluded from the Analytic Sample	
	Unweighted n	Weighted % (95% CI)	Unweighted n	Weighted % (95% CI)	Unweighted n	Weighted % (95% CI)	Unweighted n	Weighted % (95% CI)	Unweighted n	Weighted % (95% CI)
**Overall**	9385	100	3423	46.6 (45.1, 48.0)	2269	26.5 (25.2, 27.7)	2669	27.0 (25.7, 28.2)	1024	100
**Age**										
18-24	310	10.3 (9.2, 11.5)	108	9.5 (7.7, 11.3)	58	8.9 (6.5, 11.2)	66	8.7 (6.5, 10.9)	78	15.8 (12.4, 19.2)
25-34	856	16.8 (15.6, 18.0)	380	18.4 (16.4, 20.3)	162	12.6 (10.4, 14.9)	137	11.3 (9.2, 13.5)	177	24.8 (21.3, 28.4)
35-44	1252	18.5 (17.4, 19.6)	431	17.3 (15.6, 19.1)	310	19.9 (17.5, 22.2)	306	16.1 (14.1, 18.1)	205	22.2 (19.1, 25.4)
45-54	1255	14.3 (13.4, 15.2)	411	13.3 (11.9, 14.7)	318	15.3 (13.4, 17.1)	370	15.2 (13.4, 17.0)	156	13.9 (11.5, 16.3)
55-64	2043	17.6 (16.7, 18.5)	662	16.8 (15.3, 18.3)	544	20 (18.1, 21.9)	653	21.5 (19.5, 23.4)	184	11.9 (9.8, 13.9)
65-74	2342	14.5 (13.8, 15.3)	898	15.8 (14.5, 17.0)	563	14.9 (13.4, 16.4)	726	17.7 (16.1, 19.3)	155	7.6 (6.2, 9.1)
75+	1327	8.0 (7.4, 8.5)	533	8.9 (7.9, 9.8)	314	8.4 (7.2, 9.7)	411	9.5 (8.3, 10.7)	69	3.8 (2.7, 4.8)
**Gender**										
Female	3866	51.1 (49.7, 52.5)	1406	48.6 (46.4, 50.9)	865	48.1 (45.2, 50.9)	946	46.5 (43.9, 49.2)	649	65.3 (61.6, 69.1)
Male	5340	47.4 (46.0, 48.7)	1930	49.0 (46.8, 51.3)	1368	51.3 (48.4, 54.1)	1690	52.2 (49.5, 54.9)	352	33.2 (29.5, 36.9)
Other	125	1.6 (1.2, 2.0)	74	2.3 (1.6, 3.0)	22	0.7 (0.3, 1.1)	14	1.3 (0.4, 2.2)	15	1.5 (0.5, 2.5)
**Race and ethnicity**										
White, Non-Hispanic	7014	62.7 (61.2, 64.1)	2353	57.1 (54.8, 59.3)	1808	71.2 (68.3, 74.1)	2281	77.9 (75.2, 80.5)	572	45.8 (42.1, 49.6)
Black, Non-Hispanic	748	12.0 (10.9, 13.0)	378	14.5 (12.8, 16.2)	153	10.9 (8.7, 13.1)	63	5.1 (3.6, 6.7)	154	16.2 (13.3, 19.2)
Hispanic, any race	1016	16.9 (15.7, 18.1)	418	18.6 (16.6, 20.6)	196	12.3 (10.2, 14.5)	192	9.9 (8.2, 11.7)	210	27.1 (23.4, 30.7)
American Indian or Alaska Native, Non-Hispanic	47	1.1 (0.7, 1.5)	12	0.6 (0.1, 1.0)	10	1.1 (0.3, 1.9)	22	2.1 (0.9, 3.4)	3	0.9 (0.0, 1.8)
Asian American / Pacific Islander, Non-Hispanic	277	5.5 (4.7, 6.2)	155	7.2 (5.9, 8.5)	36	2.9 (1.7, 4.1)	34	2.8 (1.6, 4.0)	52	8.2 (5.7, 10.8)
Some other race, Non-Hispanic	19	0.1 (0.1, 0.2)	9	0.1 (0.0, 0.3)	3	0.1 (0.0, 0.2)	5	0.3 (0.0, 0.6)	2	0.0 (0.0, 0.1)
2+ Races, Non-Hispanic	264	1.8 (1.4, 2.2)	98	2.0 (1.3, 2.6)	63	1.5 (0.7, 2.2)	72	1.8 (1.2, 2.5)	31	1.7 (0.9, 2.6)
**Marital status**										
Now married	5961	56.2 (54.8, 57.6)	2124	56.2 (54.0, 58.5)	1510	61.3 (58.4, 64.1)	1779	58.9 (56.2, 61.6)	548	47.0 (43.3, 50.8)
Widowed	582	3.9 (3.5, 4.4)	194	3.6 (2.9, 4.2)	151	4.4 (3.4, 5.4)	180	4.5 (3.6, 5.4)	57	3.5 (2.3, 4.8)
Divorced or Separated	1132	9.6 (8.9, 10.3)	377	8.5 (7.4, 9.6)	278	10.6 (9.1, 12.1)	358	11.8 (10.3, 13.3)	119	8.1 (6.4, 9.8)
Never married	1710	30.2 (28.8, 31.6)	728	31.7 (29.4, 33.9)	330	23.7 (20.9, 26.6)	352	24.8 (22.0, 27.6)	300	41.3 (37.4, 45.2)

Table S1, continued.

Characteristic	All Wave 2 Respondents		Included in the Analytic Sample (n = 8361) How Much Do You Approve or Disapprove of…the National Rifle Association? Do Not Approve		Somewhat Approve		Strongly/Very Strongly Approve		Excluded from the Analytic Sample	
	Unweighted n	Weighted % (95% CI)	Unweighted n	Weighted % (95% CI)	Unweighted n	Weighted % (95% CI)	Unweighted n	Weighted % (95% CI)	Unweighted n	Weighted % (95% CI)
**Education**										
No high school diploma or GED	416	9.5 (8.4, 10.5)	112	6.9 (5.5, 8.3)	75	7.7 (5.7, 9.7)	103	7.8 (6.0, 9.6)	126	19.0 (15.6, 22.3)
High school graduate (diploma, GED)	2002	28.2 (26.9, 29.6)	535	21.9 (19.9, 23.9)	433	24.1 (21.6, 26.6)	695	35.5 (32.8, 38.1)	339	37.7 (33.9, 41.4)
Some college or Associate's degree	2773	27.1 (25.9, 28.3)	843	23.8 (21.9, 25.7)	758	32.2 (29.6, 34.8)	901	31.3 (29.0, 33.7)	271	22.9 (19.9, 25.9)
Bachelor's degree	2337	20.1 (19.1, 21.1)	967	24.6 (22.8, 26.4)	573	20.9 (18.8, 22.9)	620	16.9 (15.2, 18.5)	177	13.6 (11.1, 16.0)
Master's degree or higher	1857	15.1 (14.2, 15.9)	966	22.8 (21.1, 24.4)	430	15.2 (13.3, 17.0)	350	8.5 (7.4, 9.6)	111	6.9 (5.4, 8.5)
**Household Income**										
Less than $10,000	233	3.9 (3.2, 4.5)	61	2.0 (1.4, 2.7)	42	3.6 (2.2, 5.0)	67	4.1 (2.9, 5.3)	63	7.8 (5.5, 10.1)
$10,000 to $24,999	727	8.9 (8.1, 9.8)	235	7.2 (6.0, 8.3)	161	8.5 (6.7, 10.2)	201	9.1 (7.4, 10.8)	130	13.1 (10.4, 15.7)
$25,000 to $49,999	1617	17.0 (15.9, 18.0)	519	14.7 (13.1, 16.3)	372	15.0 (13.1, 16.9)	505	18.7 (16.6, 20.7)	221	21.9 (18.7, 25.1)
$50,000 to $74,999	1631	16.3 (15.3, 17.4)	515	14.9 (13.3, 16.5)	428	17.7 (15.5, 19.9)	518	17.6 (15.6, 19.6)	170	16.3 (13.4, 19.1)
$75,000 to $99,999	1499	13.2 (12.3, 14.1)	563	13.9 (12.4, 15.3)	374	13.4 (11.7, 15.1)	433	13.5 (11.8, 15.2)	129	11.3 (9.0, 13.6)
$100,000 to $149,999	1734	17.9 (16.8, 18.9)	659	18.6 (16.9, 20.4)	435	20.2 (17.9, 22.4)	489	17.6 (15.6, 19.6)	151	13.9 (11.3, 16.4)
$150,000 or more	1944	22.8 (21.6, 23.9)	871	28.7 (26.7, 30.7)	457	21.7 (19.4, 24.0)	456	19.5 (17.4, 21.5)	160	15.8 (13.0, 18.5)
**Employment**										
Working - as a paid employee	4291	52.9 (51.6, 54.3)	1545	54.1 (51.9, 56.3)	1081	55.6 (52.8, 58.4)	1138	49.0 (46.4, 51.7)	527	52.0 (48.2, 55.9)
Working - self-employed	709	7.2 (6.5, 8.0)	266	6.7 (5.7, 7.6)	186	7.3 (5.9, 8.6)	197	8.5 (6.9, 10.0)	60	7.0 (4.8, 9.1)
Not working - on temporary layoff from a job	35	0.5 (0.3, 0.7)	14	0.5 (0.2, 0.9)	4	0.2 (0.0, 0.5)	11	0.5 (0.1, 1.0)	6	0.7 (0.1, 1.3)
Not working - looking for work	272	5.2 (4.4, 5.9)	98	4.9 (3.7, 6.1)	68	5.6 (3.9, 7.3)	61	3.2 (2.2, 4.2)	45	7.6 (5.2, 10.0)
Not working - retired	3367	21.3 (20.4, 22.2)	1261	22.4 (20.9, 23.9)	805	22.4 (20.5, 24.3)	1083	27.3 (25.2, 29.3)	218	10.7 (8.9, 12.4)
Not working - disabled	286	4.5 (3.9, 5.2)	93	4.1 (3.1, 5.1)	49	3.2 (2.1, 4.3)	85	4.5 (3.3, 5.7)	59	7.1 (5.0, 9.2)
Not working - other	425	8.3 (7.4, 9.2)	146	7.3 (5.9, 8.6)	76	5.7 (4.2, 7.2)	94	7.0 (5.2, 8.8)	109	15.0 (12.1, 17.9)
**Census division**										
New England	373	4.7 (4.1, 5.3)	178	6.1 (5.0, 7.2)	78	4.2 (3.0, 5.4)	88	3.5 (2.6, 4.4)	29	3.6 (2.1, 5.1)
Mid-Atlantic	1003	12.4 (11.5, 13.4)	355	12.7 (11.2, 14.2)	246	12.2 (10.4, 14.0)	262	10.2 (8.6, 11.7)	140	15.1 (12.3, 17.8)
East-North Central	1373	14.3 (13.3, 15.2)	473	13.8 (12.2, 15.3)	351	15.4 (13.4, 17.3)	413	15.3 (13.4, 17.1)	136	12.8 (10.3, 15.4)
West-North Central	679	6.4 (5.7, 7.0)	229	5.7 (4.8, 6.7)	176	6.9 (5.6, 8.3)	219	8.3 (6.9, 9.7)	55	4.6 (3.1, 6.2)
South Atlantic	1874	20.5 (19.3, 21.6)	677	20.4 (18.6, 22.2)	466	20.1 (17.9, 22.3)	539	21.2 (18.9, 23.4)	192	20.1 (17.0, 23.2)
East-South Central	540	5.9 (5.2, 6.5)	158	4.7 (3.7, 5.7)	121	5.9 (4.4, 7.4)	197	7.5 (6.2, 8.9)	64	6.3 (4.4, 8.2)
West-South Central	975	12 (11.0, 12.9)	301	10.3 (8.9, 11.7)	241	12.9 (10.8, 15.0)	322	13.2 (11.4, 15)	111	12.9 (10.0, 15.7)
Mountain	819	7.7 (7.0, 8.4)	285	7.8 (6.6, 9.0)	201	7.7 (6.3, 9.1)	259	8.7 (7.2, 10.2)	74	6..0 (4.3, 7.7)
Pacific	1749	16.3 (15.3, 17.3)	767	18.4 (16.8, 20.0)	389	14.7 (12.7, 16.7)	370	12.3 (10.6, 14)	223	18.5 (15.7, 21.4)

Table S2. Sociodemographic characteristics (unweighted) of respondents and non-respondents in the 2022 and 2023 surveys

Characteristic	2022 (Wave1)				Wave1 respondents who left the panel prior to wave2 (n = 1,807)		2023 (Wave2)			
	Respondents (n = 12,947)		Non-respondents (n = 8,318)				Respondents (n = 9,385)		Non-respondents (n = 1,755)	
	Unweighted n	Unweighted %	Unweighted n	Unweighted %	Unweighted n	Unweighted %	Unweighted n	Unweighted %	Unweighted n	Unweighted %
Age										
18-24	488	3.8	1059	12.7	86	4.8	310	3.3	92	5.2
25-34	1309	10.1	1411	17.0	210	11.6	856	9.1	243	13.8
35-44	1884	14.6	1732	20.8	326	18	1252	13.3	306	17.4
45-54	1847	14.3	1599	19.2	335	18.5	1255	13.4	257	14.6
55-64	2794	21.6	1254	15.1	391	21.6	2043	21.8	360	20.5
65-74	2952	22.8	861	10.4	313	17.3	2342	25.0	297	16.9
75+	1673	12.9	402	4.8	146	8.1	1327	14.1	200	11.4
Non-response	0	0.0	0	0.0	0	0.0	0	0.0	0	0.0
Gender										
Male	7158	55.3	3993	48.0	854	47.3	5437	57.9	867	49.4
Female	5789	44.7	4325	52.0	953	52.7	3948	42.1	888	50.6
Non-response	0	0.0	0	0.0	0	0.0	0	0.0	0	0.0
Race and ethnicity										
Black, non-Hispanic	1097	8.5	1039	12.5	170	9.4	749	8.0	178	10.1
Hispanic	1504	11.6	1561	18.8	237	13.1	1016	10.8	251	14.3
White, non-Hispanic	9493	73.3	5030	60.5	1272	70.4	7014	74.7	1207	68.8
Other, non-Hispanic	499	3.9	370	4.4	77	4.3	346	3.7	76	4.3
2+ races, non-Hispanic	354	2.7	318	3.8	51	2.8	260	2.8	43	2.5
Non-response	0	0.0	0	0.0	0	0.0	0	0.0	0	0.0
Marital status										
Now married	8074	62.4	4460	53.6	1089	60.3	5961	63.5	1024	58.3
Widowed	770	5.9	303	3.6	82	4.5	582	6.2	106	6
Divorced	1456	11.2	858	10.3	240	13.3	1010	10.8	206	11.7
Separated	193	1.5	191	2.3	34	1.9	122	1.3	37	2.1
Never married	2454	19.0	2506	30.1	362	20	1710	18.2	382	21.8
Non-response	0	0.0	0	0.0	0	0.0	0	0.0	0	0.0
Education										
No high school diploma or GED	624	4.8	694	8.3	121	6.7	416	4.4	87	5
High school graduate or GED	2813	21.7	2084	25.1	452	25	2002	21.3	359	20.5
Some college or Associate degree	3896	30.1	2649	31.8	584	32.3	2773	29.5	539	30.7
Bachelor's degree	3133	24.2	1726	20.8	372	20.6	2337	24.9	424	24.2
Master's degree or higher	2481	19.2	1165	14.0	278	15.4	1857	19.8	346	19.7
Non-response	0	0.0	0	0.0	0	0.0	0	0.0	0	0.0

Table S2, continued.

Characteristic	2022 (Wave1)				Wave1 respondents who left the panel prior to wave2 (n = 1,807)		2023 (Wave2)			
	Respondents (n = 12,947)		Non-respondents (n = 8,318)				Respondents (n = 9,385)		Non-respondents (n = 1,755)	
	Unweighted n	Unweighted %	Unweighted n	Unweighted %	Unweighted n	Unweighted %	Unweighted n	Unweighted %	Unweighted n	Unweighted %
Household Income										
< $10,000	371	2.9	410	4.9	72	4	233	2.5	66	3.8
$10,000 - $24,999	1078	8.3	793	9.5	189	10.5	727	7.7	162	9.2
$25,000 - $49,999	2232	17.2	1558	18.7	318	17.6	1617	17.2	297	16.9
$50,000 - $74,999	2236	17.3	1427	17.2	313	17.3	1631	17.4	292	16.6
$75,000 - $99,999	1999	15.4	1203	14.5	236	13.1	1499	16.0	264	15
$100,000 - $149,999	2410	18.6	1461	17.6	336	18.6	1734	18.5	340	19.4
>= $150,000	2621	20.2	1466	17.6	343	19	1944	20.7	334	19
Non-response	0	0.0	0	0.0	0	0.0	0	0.0	0	0.0
Employment										
Working full-time	5645	43.6	4514	54.3	889	49.2	3869	41.2	887	50.5
Working part-time	1620	12.5	1342	16.1	258	14.3	1133	12.1	229	13
Not working	5682	43.9	2462	29.6	660	36.5	4383	46.7	639	36.4
Non-response	0	0.0	0	0.0	0	0.0	0	0.0	0	0.0
Census division										
New England	509	3.9	297	3.6	73	4	374	4.0	62	3.5
Mid-Atlantic	1407	10.9	915	11.0	191	10.6	1001	10.7	215	12.3
East-North Central	1878	14.5	1117	13.4	262	14.5	1370	14.6	246	14
West-North Central	952	7.4	597	7.2	137	7.6	676	7.2	139	7.9
South Atlantic	2538	19.6	1652	19.9	326	18	1881	20.0	331	18.9
East-South Central	737	5.7	579	7.0	117	6.5	538	5.7	82	4.7
West-South Central	1371	10.6	1093	13.1	207	11.5	965	10.3	199	11.3
Mountain	1125	8.7	573	6.9	156	8.6	825	8.8	144	8.2
Pacific	2430	18.8	1495	18.0	338	18.7	1755	18.7	337	19.2
Non-response	0	0.0	0	0.0	0	0.0	0	0.0	0	0.0

Mean (SD) ages were as follows: Wave 1 responders, 55.7 (16.7); Wave 1 non-responders, 45.4 (16.8); Wave 1 respondents who left the panel prior to Wave 2, 52.2 (16.2); Wave 2 responders, 57.0 (16.5); Wave 2 non-responders, 52.5 (17.5).

This table previously appeared in the supplement to Wintemute GJ, Robinson SL, Crawford A, Tomsich EA, Reeping PM, Shev AB, Velasquez B, Tancredi D. Single-year change in views of democracy and society and support for political violence in the USA: findings from a 2023 nationally representative survey. Injury Epidemiology. 2024 May 21;11:20.
